# Strategies for overcoming challenges in selective electrochemical CO_2_ conversion to ethanol

**DOI:** 10.1016/j.isci.2024.110437

**Published:** 2024-07-02

**Authors:** Zihong Wang, Yecheng Li, Zhihao Ma, Dazhuang Wang, Xiaodi Ren

**Affiliations:** 1 School of Chemistry and Materials Science, University of Science and Technology of China, Anhui 230026, China

**Keywords:** chemical engineering, chemistry, electrochemical engineering, electrochemistry

## Abstract

The electrochemical conversion of carbon dioxide (CO_2_) to valuable chemicals is gaining significant attention as a pragmatic solution for achieving carbon neutrality and storing renewable energy in a usable form. Recent research increasingly focuses on designing electrocatalysts that specifically convert CO_2_ into ethanol, a desirable product due to its high-energy density, ease of storage, and portability. However, achieving high-efficiency ethanol production remains a challenge compared to ethylene (a competing product with a similar electron configuration). Existing electrocatalytic systems often suffer from limitations such as low energy efficiency, poor stability, and inadequate selectivity toward ethanol. Inspired by recent progress in the field, this review explores fundamental principles and material advancements in CO_2_ electroreduction, emphasizing strategies for ethanol production over ethylene. We discuss electrocatalyst design, reaction mechanisms, challenges, and future research directions. These advancements aim to bridge the gap between current research and industrialized applications of this technology.

## Graphical abstract

**Figure:**
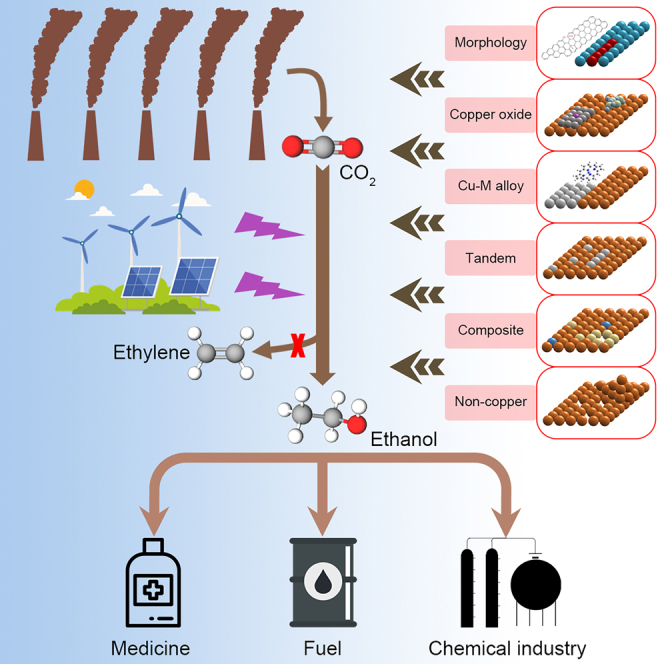

## Introduction

**Figure 1:**
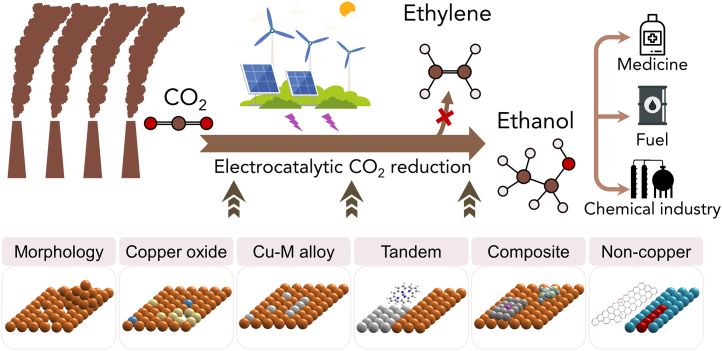
Schematic of strategies for selective ethanol over ethylene production in electrochemical CO_2_ reduction and its application

Electrocatalytic carbon dioxide (CO_2_) reduction reaction (CO_2_RR) is one of the most promising routes toward achieving carbon neutrality.^1^^,^^2^^,^^3^ This field has evoked a plethora of research activities, primarily focusing on upgrading CO_2_ into building-block chemicals and liquid fuels.^4^ During the CO_2_RR, the applied electrical energy is converted to stored chemical energy via reorganizing the molecular bonds in CO_2_ and water to generate reduction products in particular high-value multi-carbon (C_2+_) olefins and oxygenates through copper (Cu) base catalysis.^5^^,^^6^^,^^7^ These products play crucial roles in the current supply of energy and chemicals such as fuel additives, plastics, disinfectants, and pharmaceuticals.^8^^,^^9^ Among the various C_2+_ products formed on Cu catalysts, ethanol stands out as a compelling target among the myriad of products achievable through CO_2_RR due to its substantial market power and wide market demand (over USD 137.8 billion in 2030).^10^^,^^11^^,^^12^^,^^13^^,^^14^ Its attractiveness stems from its versatile applications, serving not only as a solvent in chemical synthesis but also as a vital chemical feedstock for producing various organic compounds like acetaldehyde, acetic acid, diethyl ether, and so on (Figure 1). Beyond industrial uses, ethanol finds valuable applications in medicine, where it functions as an antiseptic and disinfectant. Moreover, it offers the added advantage of being a renewable fuel, possessing an impressive energy density of 26.8 MJ kg^−1^, making it a highly promising option for sustainable energy solutions.^15^ However, ethylene is usually the major C_2+_ product during the Cu-based electrocatalytic CO_2_RR.^16^ Although ethanol can also be formed, the ratio of Faradaic efficiencies (FEs) of ethanol to ethylene is typically lower than 0.5 on a Cu-based catalyst.^4^^,^^10^ Therefore, the development of an efficient CO_2_RR reduction product conversion system from ethylene to ethanol plays an important role in our understanding of the intermediate process of the reaction and the identification of the nature of product differences.

In CO_2_RR, ethanol and ethylene are both 12-electron reduced products, and similar equilibrium potential (ethanol is 0.084 V and ethylene is 0.060 V vs. reversible hydrogen electrode [RHE]). Besides, it is believed that they are derived from a shared key intermediate (∗CHCOH, the asterisk represents adsorption), and that ethylene is generated after C−O bond-breaking from ∗CHCOH. Thus, ethanol and ethylene are the two mainly competing C_2_ products.^17^ A technoeconomic analysis of CO_2_RR systems shows that C_2+_ production can become profitable only once the partial current density exceeds 100 mA cm^−2^. Recently, CO_2_RR to ethylene has been reported with an FE over 80% with a partial current density of 300 mA cm^−2^ or an FE over 60% with a partial current density of 1.2 A cm^−2^.^18^^,^^19^ Unfortunately, the best ethanol FE reported so far is 60% with a partial current density of 300 mA cm^−2^ or FE is 40% with a partial current density of 320 mA cm^−2^.^20^^,^^21^ According to the current research progress, there is a serious mismatch in the reduction efficiency of the two C_2+_ reduction products of ethanol and ethylene, which is mainly due to the following reasons. There are many studies have shown that the selectivity toward the production of ethylene on the Cu metal surface is often higher than ethanol because the former exhibits thermodynamically favored formation.^22^^,^^23^ More importantly, due to its more saturated structure in comparison to ethylene, the stabilization of next-intermediates for ethanol on a pure Cu surface is inherently more challenging.^20^ As a result, the production of ethanol through C−O bond-reserving of ∗HCCOH faces significant chemical difficulties, making it less competitive against ethylene generation.^24^^,^^25^ Consequently, the production of ethylene on Cu-based catalysts is typically 2–3 times higher than that of ethanol.^26^^,^^27^^,^^28^^,^^29^ An urgent requirement arises to devise an efficient strategy enabling the selective inversion of isoelectronic C_2+_ products (ethanol and ethylene), along with a clear delineation of the factors influencing this conversion. Such efforts will enhance understanding of the underlying principles governing the preparation of symmetric and asymmetric products.

Developing electrocatalysts with high activity, selectivity, and energy efficiency toward ethanol is still a wide-open question. Advances in fundamental understanding in recent years have led to new material designs that significantly improve the CO_2_-to-ethanol conversion.^30^^,^^31^^,^^32^^,^^33^^,^^34^ Inspired by these works, herein we provide a comprehensive review of the fundamental aspects of CO_2_RR to ethanol from both mechanistic insights and experimental findings. Different from previous reviews on ethanol production, this review sets itself apart by ethanol enhancement strategies with emphasis on the reciprocal relationship between ethylene and ethanol, which are isoelectronic entities. Our aim is to highlight strategies for enhancing the efficiency of the isoelectronic C_2_ product selective inversion between ethanol and ethylene, which have demonstrated tremendous potential in transforming the CO_2_RR chemistry. In this review, we delve into key developments that facilitate the isoelectronic C_2+_ product selective inversion of ethanol and ethylene within the catalyst design classification (Figure 1). Our discussion is grounded in the following representative examples, the design of pure Cu catalyst surface morphology, the architecture of catalyst surface atoms, and the construction of modulator and tandem structure, as well as metal free catalytic materials. Furthermore, we present a thorough overview of the underlying reaction mechanism, shedding light on the intricacies and factors governing this transformation. We then highlight the remaining challenges and provide an outlook on promising research directions.

## Ethanol versus ethylene mechanism

The generation of various types of C_2_ products follows specific mechanisms, which are influenced by the complexity and diversity of their reaction processes.^35^ Understanding the reaction mechanism of a single C_2_ product pose a significant challenge.^9^ However, it’s important to note that the production of C_2_ products typically involve a key step: the C−C coupling between ∗CO. This step forms the basis for different pathways that ultimately yield diverse products.^36^ According to the Sabatier principle, optimal catalysts bind atoms and molecules with just the right strength-not too weak to activate the reactants, yet not too strong to allow for the desorption of the products.^4^ However, the selectivity toward C_2+_ products can not only be explained by the binding energy of carbon monoxide (CO). In the quest to design efficient catalysts for the reduction of CO_2_ to ethanol, it’s crucial to comprehend the mechanistic features of ethanol formation. This is because the C_2_ selectivity is usually biased toward ethylene rather than ethanol. Predicted reaction mechanisms for C_2_ products are highly complex and subject to variation due to factors such as the catalytic surface, environmental conditions, computational methods, and so on.^37^ Therefore, this section aims to introduce several perspectives to provide a comprehensive understanding of the subject. Prior studies and computations have established that C−C coupling forms the foundation for the production of ethanol and ethylene.^38^ Subsequent to this, variations in the proton coupling electron transfer process and the dehydration process of intermediates have led to divergent paths for the final products. To gain a clearer understanding of the reaction pathway, we categorize the common intermediates that result from CO2 in the generation of ethanol and ethylene into two primary types: oxygen and carbon adsorption and carbon adsorption. The type of adsorption also influences the subsequent reaction mechanisms and enhancement strategies. We will discuss these two types separately in the following sections.^36^^,^^39^

### Oxygen and carbon adsorption intermediate pathways

**Figure 2:**
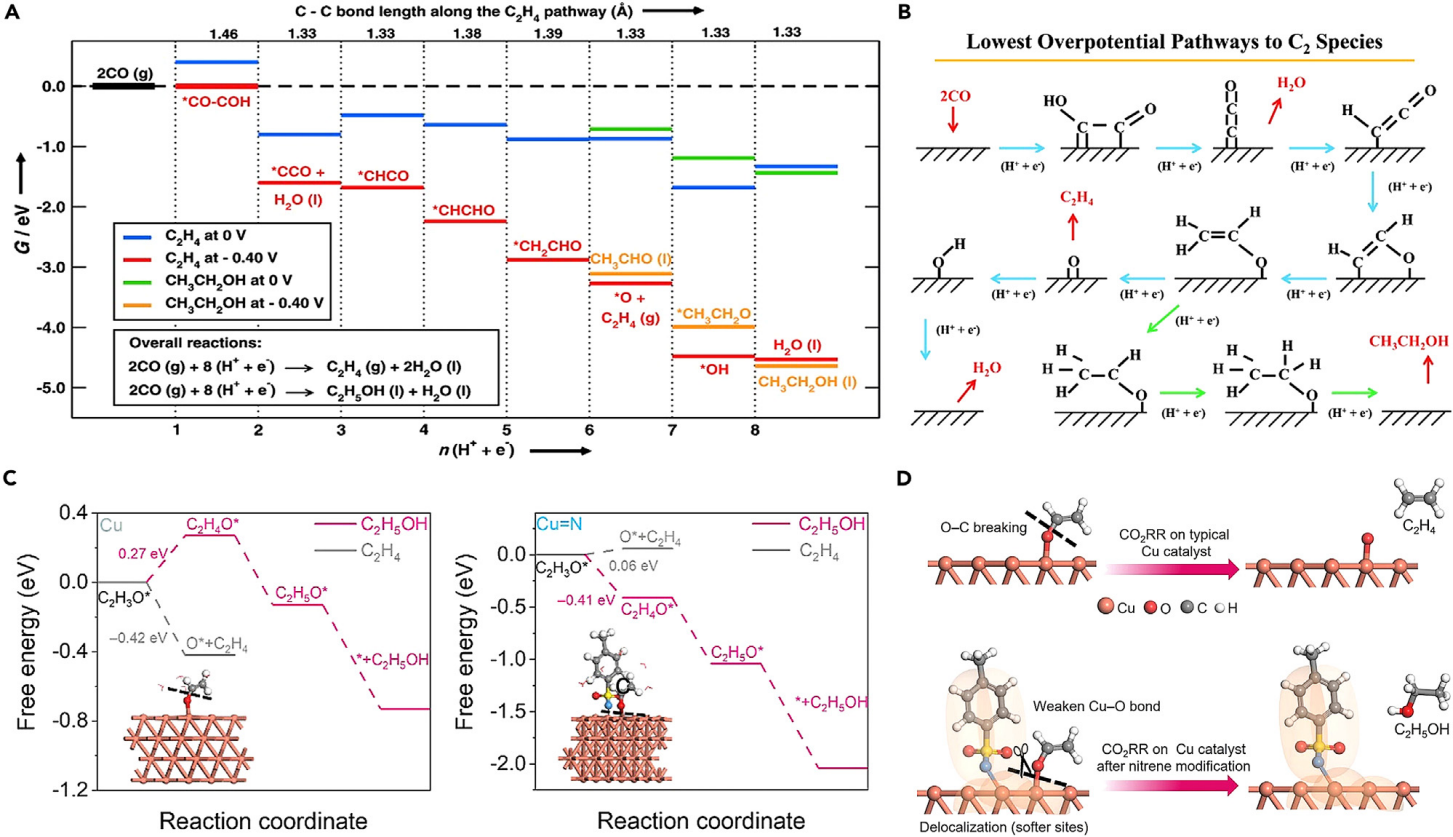
Oxygen adsorption intermediate pathways of CO_2_ to ethanol (A) Lowest overpotential pathways for the electroreduction of CO to ethanol and ethylene. The potential-determining step is the first proton-electron transfer. The energy levels are given at U = 0 V vs. RHE and U = −0.4 V, which is the calculated onset potential.^40^ Copyright 2013, Wiley-VCH. (B) Schematic representations of the species involved in the pathways to C_2_H_4_ (blue) and MeCHO/EtOH (green).^40^ Copyright 2013, Wiley-VCH. (C) Energy diagrams of hydrogenating the ∗OC_2_H_3_ intermediate to produce ethylene and ethanol on Cu and Cu=N models.^41^ Copyright 2024, American Chemical Society. (D) During CO_2_ reduction, CH_2_ = CHO adsorbs on Cu, leading to either ethylene or ethanol. On regular Cu, lower O−C bond energy favors ethylene formation. On nitrene-doped Cu, altered electron distribution weakens the O−Cu bond, increasing ethanol selectivity.^41^ Copyright 2024, American Chemical Society.

Koper and colleagues proposed a detailed reaction mechanism to deeply investigate the process by which CO generates C_2_ species such as ethylene (C_2_H_4_), ethanol (C_2_H_5_OH), and acetaldehyde (MeCHO) through an electrochemical reduction reaction on the Cu (100) electrode.^40^ This mechanism involves the coupling of two CO molecules into a ∗C_2_O_2_ intermediate via electron transfer. This is then followed by the generation of the target C_2_ species through a proton-electron transfer reaction. The study utilized a computational hydrogen electrode model to calculate the adsorption energy and determine the onset potential of the reaction. Figures 2A and 2B show pathways for CO reduction to C_2_H_4_, MeCHO, and C_2_H_5_OH. ∗COCHO is less stable than ∗CO−COH by 0.16 eV (Figure 2A), favoring O atom hydrogenation in ∗C_2_O_2_. Thus, ∗CO−COH is used as the starting intermediate after dimerization. This intermediate undergoes five hydrogenation steps to form ∗CH_2_CHO, where the pathways diverge (Figure 2B). Ethylene forms through the cleavage of the remaining C−O bond and desorbs, leaving an adsorbed oxygen atom (∗O), which is further hydrogenated to form water and desorbs. Alternatively, MeCHO forms by protonation of the α-carbon in ∗CH_2_CHO. MeCHO then reduces to C_2_H_5_OH via an ethoxy intermediate (∗CH_3_CH_2_O) through two more hydrogenation steps. Calculations show that in the sixth step, the energy required to generate ethylene is about 0.2 eV lower than that for ethanol, corroborating empirical results that on a Cu electrode, the FE for ethanol is generally lower than for ethylene (Figure 2A). Informed by this reaction mechanism, Zheng and his team proposed that by weakening the Cu−O interaction to be less than the O−C interaction, the product selectivity might shift from ethylene to ethanol by changing the sequence of bond breaking (Figure 2D).^41^ Given that Cu−O forms a Lewis acid-base pair, and in line with the hard-soft acid-base theory, the oxygen atom in CH_2_CHO∗ is a hard base that is not inclined to bind to a soft acid. By adjusting the Cu site to a softer state (i.e., more electron delocalized), its interaction with oxygen may be weakened, promoting Cu−O bond breaking and the subsequent ethanol formation pathway. For Cu, the energy barrier for hydrogenating CH_2_CHO∗ to form CH_3_CHO∗ is 0.27 eV, which is higher than that for forming C_2_H_4_ + ∗O (−0.42 eV), indicating a preference for the C_2_H_4_ formation pathway. Conversely, for a CuN catalyst with improved electron delocalization capabilities, the energy barrier for hydrogenating CH_2_CHO∗ to form CH_3_CHO∗ is −0.41 eV, significantly lower than for forming C_2_H_4_ and O∗ (0.06 eV), thereby altering the product selectivity from C_2_H_4_ to C_2_H_5_OH (Figure 2C). For the intermediate pathways involving oxygen and carbon adsorption, adjusting the bond energy between M−O and C−O is a viable strategy to optimize the reaction process for ethanol production. Furthermore, enhancing the attack on the double bond C through the optimization of the hydrogenation process is also an effective approach. Pursuing a direction that does not disrupt the C−O bond holds promise as well.

### Carbon adsorption intermediate pathways

**Figure 3:**
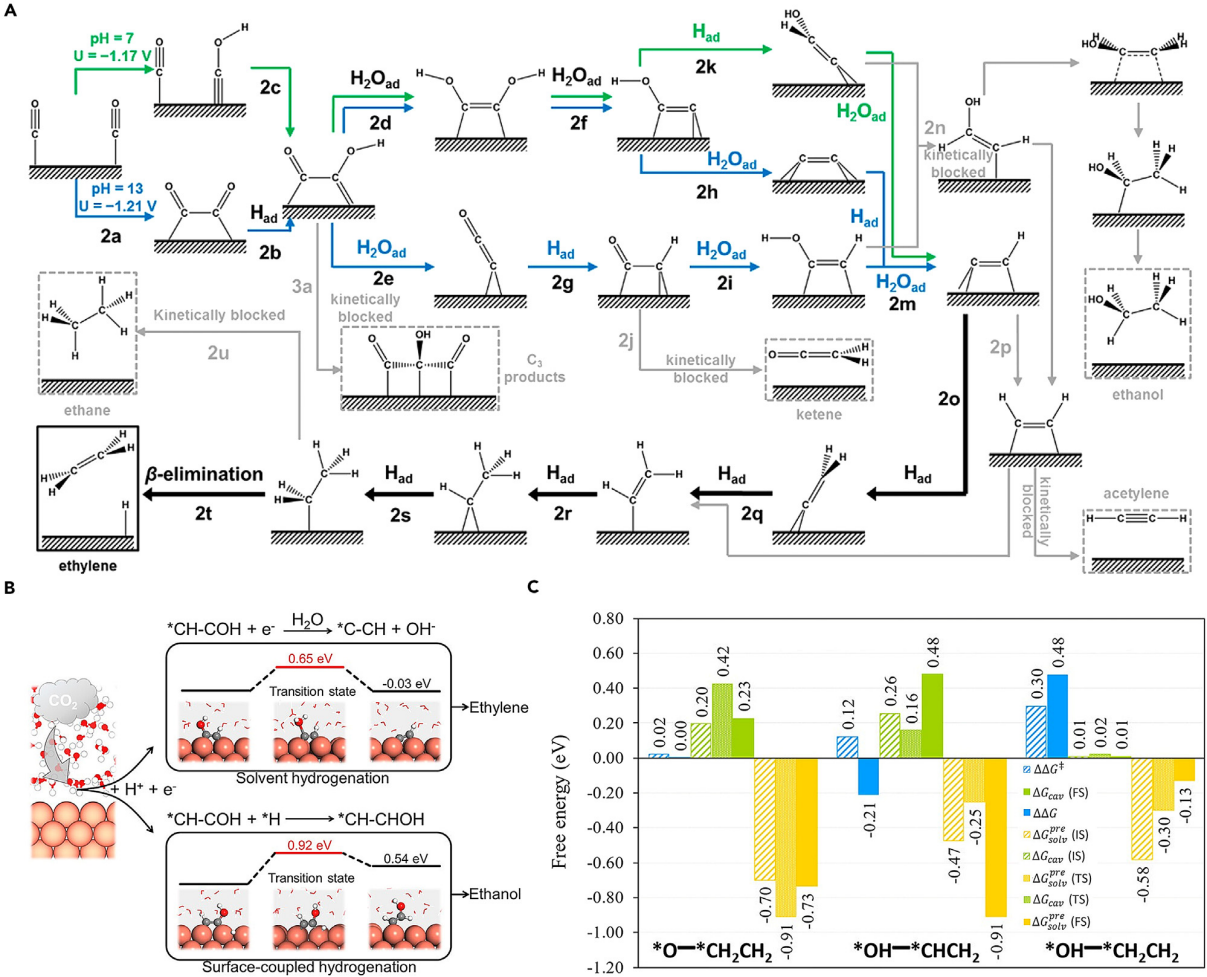
Carbon adsorption intermediate pathways of CO_2_ to ethanol (A) Predicted complete reaction pathways to C_2_ products featuring H_2_Oad mechanism.^42^ Copyright 2017, American Chemical Society. (B) The CO_2_ reduction on Cu(100) diverges into ethylene or ethanol paths.^17^ Copyright 2023, American Chemical Society. (C) Plots of changes in TS barriers (ΔG^⧧^) and changes in reaction free energy (ΔG) between those obtained from the explicit solvent model and the vacuum model for three C–O bond dissociation reactions: (i) ∗CH_2_CH_2_O → ∗O + ∗CH_2_CH_2_, (ii) ∗CH_2_CHOH → ∗OH + ∗CHCH_2_, and (iii) ∗CH_2_CH_2_OH → ∗OH + ∗CH_2_CH_2_, and plots of ΔG_cav_ and ΔG_solv_^pre^ of IS, TS, and FS structures of the three reactions.^43^ Copyright 2021, American Chemical Society.

Xiao et al. explored the atomic-level mechanism of CO electrochemical reduction (CORR) on the copper (111) surface to generate C_1_ and C_2_ products. Using grand canonical quantum mechanics calculations and considering the solvation effect, their research revealed the mechanisms behind the production of ethylene and ethanol.^42^ They proposed CO−COH as a new coupling mechanism. The results show that surface-bound water is crucial in forming different intermediates from CO−COH through various hydrogenation processes (Figure 3A). After CO−COH undergoes three hydrogenation steps, two key intermediates are formed: ∗C=(CH)OH under neutral conditions and ∗CH = COH under alkaline conditions, both capable of leading to ethanol. For the ∗CH = COH intermediate, dehydration with H_2_O∗ forms ∗CCH (ΔG^⧧^ = 1.16 eV), but further hydrogenation to produce ethanol via ∗CH = COH is kinetically prohibited (ΔG^⧧^ = 1.46 eV). For the ∗C=(CH)OH intermediate, H_2_O∗ drives dehydration to form ∗CH = C (ΔG^⧧^ = 0.86 eV) also blocks the hydrogenation pathway to ethanol (ΔG^⧧^ = 1.13 eV). Therefore, “hot” surface water is the key in preventing ethanol production. This suggests that destabilizing the H_2_O∗ pathway could promote ethanol production. In addition to kinetically limiting ethanol production, the interaction between water molecules and intermediates, as well as the solvation energy and solvation recombination energy with intermediates, are key factors influencing product distribution. Hirunsit and colleagues have conducted a comprehensive exploration of the various mechanisms of CO_2_RR to produce ethylene and ethanol on the Cu (100) surface, using density functional theory (DFT) calculations in explicit solvent and vacuum models.^43^ The authors have calculated that water molecules can stabilize each intermediate to varying degrees. This is evidenced by the required reorganization energy of the solvent molecule (ΔG_cav_) and the strength of the interaction between the adsorbate and the reorganization solvation shell (ΔG_solv_^pre^). For C−O bond dissociation, the degree of interaction strength between water and the intermediate (ΔG_solv_^pre^) has a greater impact on the energy barrier. The extent to which water helps stabilize the intermediate generally follows this order: ∗CH_x_−CH_x_ < ∗CH_x_CO < ∗C−CH_x_ < ∗CH_x_CH_x_O < ∗CH_x_CHOH. Water molecules play a pivotal role in whether the key different intermediates ∗CH_2_CH_2_O, ∗CH_2_CHOH, and ∗CH_2_CH_2_OH undergo C−O bond breakage (Figure 3C). For the reaction mechanism to generate ethanol, maintaining the C−O bond is crucial. Therefore, whether hydrogenation targets the carbon (C) or oxygen (O) atom is a key factor in determining the product distribution. Wang et al. elucidated the mechanism for improving ethanol selectivity on modified Cu catalysts using an explicit solvent model combined with slow growth molecular dynamics.^17^ Enhanced sampling ab initio molecular dynamics (AIMD) calculations show that the ethanol and ethylene pathways are dominated by surface-coupled hydrogenation and solvent hydrogenation of the key intermediate ∗CH−COH, respectively. For ethylene synthesis (R1), ∗CH−COH captures hydrogen from solvent water via the Eley-Rideal mechanism and dehydrates to form ∗C−CH, which is then gradually hydrogenated to generate ethylene. For ethanol synthesis (R2), the pathway begins with the coupling of ∗CH−COH and ∗H via the Langmuir-Hinshelwood mechanism to produce ∗CH−CHOH. The difference between these pathways lies in the source and target of the proton during electron transfer: solvated H^+^ or surface ∗H, and protonation on O or C. In R2, steric hindrance makes it difficult for carbon in ∗CH−COH to capture hydrogen directly from solvent water, favoring surface-coupled hydrogenation. In R1, a hydrogen bond between the hydroxyl group in ∗CH−COH and solvent water allows direct hydrogen transfer driven by electrode potential, termed solvent hydrogenation. These hydrogenation methods result in the energy required to generate ethanol being 0.57 eV higher than that for ethylene, causing ethylene to dominate the final product distribution (Figure 3B). In an effort to comprehend the source of oxygen atoms in oxygenate products resulting from CO reduction, Lum and colleagues performed a reduction of C^16^O in an H_2_^18^O electrolyte.^44^ Their findings revealed that 60−70% of the ethanol produced contained ^18^O, suggesting the oxygen originated from the solvent. Building upon prior all-solvent DFT metadynamics calculations, they factored in water incorporation and uncovered a novel mechanism. This mechanism entails a Grotthuss chain of six water molecules interacting with the ∗C−CH intermediate to yield ∗CH−CH (^18^OH), which subsequently leads to the formation of (^18^O) ethanol. This process is in competition with the formation of ethylene, which also stems from ∗C−CH. These findings corroborate the existence of multiple pathways leading to ethanol, competing with the formation of ethylene, akin to mechanisms put forth by Xiao et al., which involve shared key reaction intermediates.

## Pure copper catalysts

The conversions of CO_2_ to ethanol are multi-proton coupled electron transfer (PCET) reaction that involves in lots of intermediates and processes under the reaction conditions. The binding strength of ∗CO, a potential intermediate, to the metal surface is widely recognized as a critical factor in determining the selectivity of a metal catalyst. So far, Cu has been reported to be the efficient single-metal catalyst for the formation of C_2+_ compounds probably because of its optimal ∗CO affinity.^45^^,^^46^^,^^47^ In this section, we present a comprehensive overview of the state-of-the-art catalysts used in CO_2_-to-ethanol conversion based on pure Cu catalysts. We specifically focus on systematically examining their architecture features, from surface to interface. These features play a crucial role in stabilizing oxygenate intermediates and enabling a controllable hydrodeoxygenation step for efficient ethanol production.

### Pure copper catalyst structure design

**Figure 4:**
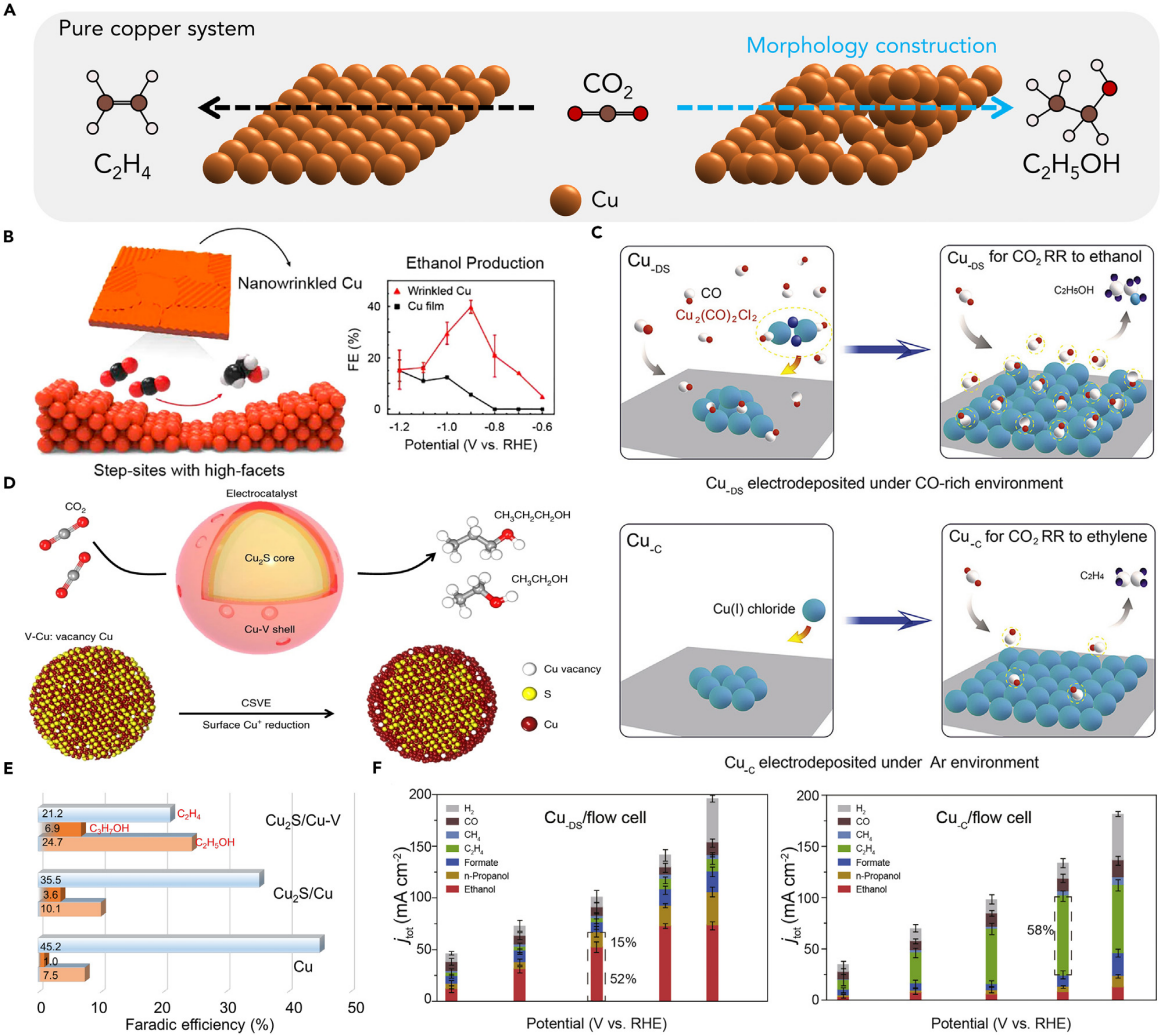
Pure Cu catalyst structure design (A) Schematic illustrating CO_2_ conversion to ethanol via optimized surface, while unoptimized copper yields ethylene. (B) Schematic of a wrinkled Cu surface and its catalytic performance for the selective conversion of CO_2_ to ethanol.^53^ Copyright 2021, American Chemical Society. (C) Cu_-DS_ catalyst synthesized under a CO-rich environment, and Cu-c catalyst deposited under an Ar atmosphere. Moreover, CO_2_RR process where the absorption density of ∗CO intermediates on catalyst surface tunes CO_2_RR selectivity of Cu_-DS_ toward ethanol or Cu_-C_ toward ethylene.^52^ Copyright 2021, Cell Press. (D) Illustrating the Cu_2_S-Cu-V CSVE electrocatalyst design through a schematic, enabling the efficient production of multi-carbon alcohols via CO_2_RR.^51^ Copyright 2018, Springer Nature. (E) FE of ethanol, propanol, and ethylene on different catalysts measured in 1M KOH electrolyte at an applied potential of −0.92 V vs. RHE in flow-cell system.^51^ Copyright 2018, Springer Nature. (F) Flow cells: current densities and product distributions under different potentials and corresponding faradaic efficiencies produced by Cu_-DS_ and Cu_-C_.^52^ Copyright 2021, Cell Press.

To date, the primary approach for ethanol-selective on pure Cu-based electrocatalysts have centered around nanostructure control tuning strategies. These strategies entail precise control of feature shape,^48^^,^^49^ dimension,^50^ atomic defects,^51^^,^^52^ and high-index facets,^53^ making them particularly intriguing for their demonstrated effectiveness in CO_2_-to-ethanol conversion performance, as evidenced by numerous previous studies.^54^ Recent research has shown that CO_2_ conversion tendencies in ethanol-selective on pure Cu-based electrocatalysts can be finely tuned by adjusting the size of Cu nanoparticles (2–15 nm range). This size variation leads to modifications in the exposed coordination number on the surface Cu atom.^50^ Moreover, the presence of atomic steps on the (100) terrace enhances ethanol productivity, while the Cu (100) facet exhibits higher ethylene selectivity compared to the Cu (111) facet.^48^^,^^55^ These findings highlight the importance of nanostructure morphology in achieving tunable selectivity for C_2+_ formation. Additionally, introducing metal atomic vacancy defects can significantly influence the electrocatalytic performance by adjusting the electronic structure of neighboring atoms, affecting the energy barriers of rate-limiting reaction intermediates. These discoveries offer valuable insights into designing efficient and selective Cu-based electrocatalysts for CO_2_ conversion to valuable ethanol instead of competitive product of ethylene. Therefore, for CO_2_-to-ethanol conversion, it is crucial to develop the special surface structure of Cu-based catalyst, different from that of ethylene (Figure 4A).

Influenced by the insights and findings from these studies, a series of recent research has extensively explored Cu surface morphology modulation. The researchers aim to overcome the constraints of conventional low currents, elevate the local current density of ethanol to industrial levels, and achieve a more profound comprehension of the reduction to ethanol rather than ethylene. These efforts seek to pave the way for enhanced electrocatalytic performance in CO_2_ conversion to valuable ethanol. Many studies have focused on Cu (100) surfaces in their exploration of ethanol products. While numerous single-crystal studies and simulations suggest that Cu (100) surfaces abundant in steps are conducive to the conversion into ethanol products, the majority of research remains confined to low-index (100) facets or low-density step-sites resulting from surface defects. To address this, Kim et al. developed a mass-producible wrinkled Cu catalyst with high-index facets through a chemical vapor deposition (CVD) graphene growth process (Figure 4B). Unlike existing methods, this catalyst consists of a high density of step-sites with unique atomic arrangements, including the (200) and (310) facets. The wrinkled Cu film exhibited remarkable ethanol selectivity, achieving a FE of 40% at −0.9 V vs. RHE. DFT calculations attributed this high ethanol productivity mainly to the (310) facet of the wrinkles, which presented a low C−C coupling barrier of 0.5 eV and a preferred reaction path toward ethanol over other products.^53^ This work emphasizes the point that optimizing catalytic material morphology allows us to reduce the energy of C−C coupling between ∗CO and select the ethanol production path. ∗CO serves as a crucial intermediate in CO_2_RR, where its surface coverage impacts the probability of C−C coupling, bonding energy, and the overall reaction path. This modulation promotes the transformation of CO_2_ toward ethanol products instead of ethylene products. Inspired by this, Zheng et al. demonstrated a novel electrochemical deposition method that leverages a CO-rich environment to create Cu catalysts with abundant defective sites, enabling selective CO_2_ electroreduction to ethanol (Figure 4C).^52^ The ∗CO-rich conditions promote the formation of defect-rich Cu surfaces by stabilizing the surface energy, which significantly enhances the CO_2_-to-ethanol reduction pathways. Through operando spectroscopies and theoretical calculations, the researchers unveiled how these defect-rich surfaces facilitate local ∗CO production, leading to increased surface ∗CO coverage and improved CO_2_-to-ethanol selectivity. In a flow-cell system, the Cu_-DS_ electrocatalyst (rich in defect sites) achieved an ethanol partial current density surpassing 100 mA cm^−2^, with a corresponding FE of 52%. In contrast, the non-defective Cu surface led to ethylene production exceeding 55% (Figure 4F). This highlights the efficient transition between ethylene and ethanol products. These outstanding results demonstrate the potential of defect-rich Cu electrocatalysts for efficient and durable CO_2_ electroreduction to ethanol, presenting a promising direction for advancing renewable energy conversion technologies. Diverging from the conventional CO atmosphere method to induce surface atomic defects, Zhuang et al. pursued an innovative approach by focusing on Cu sulfide structures known for their unique ability to create stable surface defects and regulate surface vacancy density. Their preliminary results demonstrated significant variations in the ethanol-to-ethylene ratio when they introduced S into the Cu catalyst. Encouraged by these findings, they conducted a comprehensive computational analysis to investigate a key rate-limiting step along the CO_2_-to-C_2+_ pathway.^51^ The computational studies revealed that a modified Cu_2_S core with Cu surface vacancies led to a favorable modulation, shifting selectivity toward ethanol relative to ethylene, which aligned with insights from prior research. Based on these promising outcomes, the researchers undertook a systematic study of S-enriched Cu and surface vacancies, resulting in the synthesis of a Cu_2_S-Cu-V (where V denotes vacancy) nanoparticle structure. This ingenious core-shell-vacancy engineering (CSVE) catalyst enabled them to effectively modify the C_2+_ reaction pathway, redirecting selectivity away from ethylene and toward ethanol (Figure 4D). This strategic approach aimed at suppressing unwanted C_2+_ products, rather than just C_1_ products, ultimately paving the way for more efficient (a partial current density of 100 mA cm^−2^) and selective (FE of 24%) CO_2_ electroreduction to ethanol (Figure 4E). These studies collectively highlight the potential of optimizing the morphology of catalytic materials to control C−C coupling energy and select the ethanol production pathway, paving the way for advancements in renewable energy conversion technologies. However, further research is required to deepen our understanding of the underlying mechanisms and optimize these processes for industrial applications.

### Oxidation state regulation of copper catalysts

**Figure 5:**
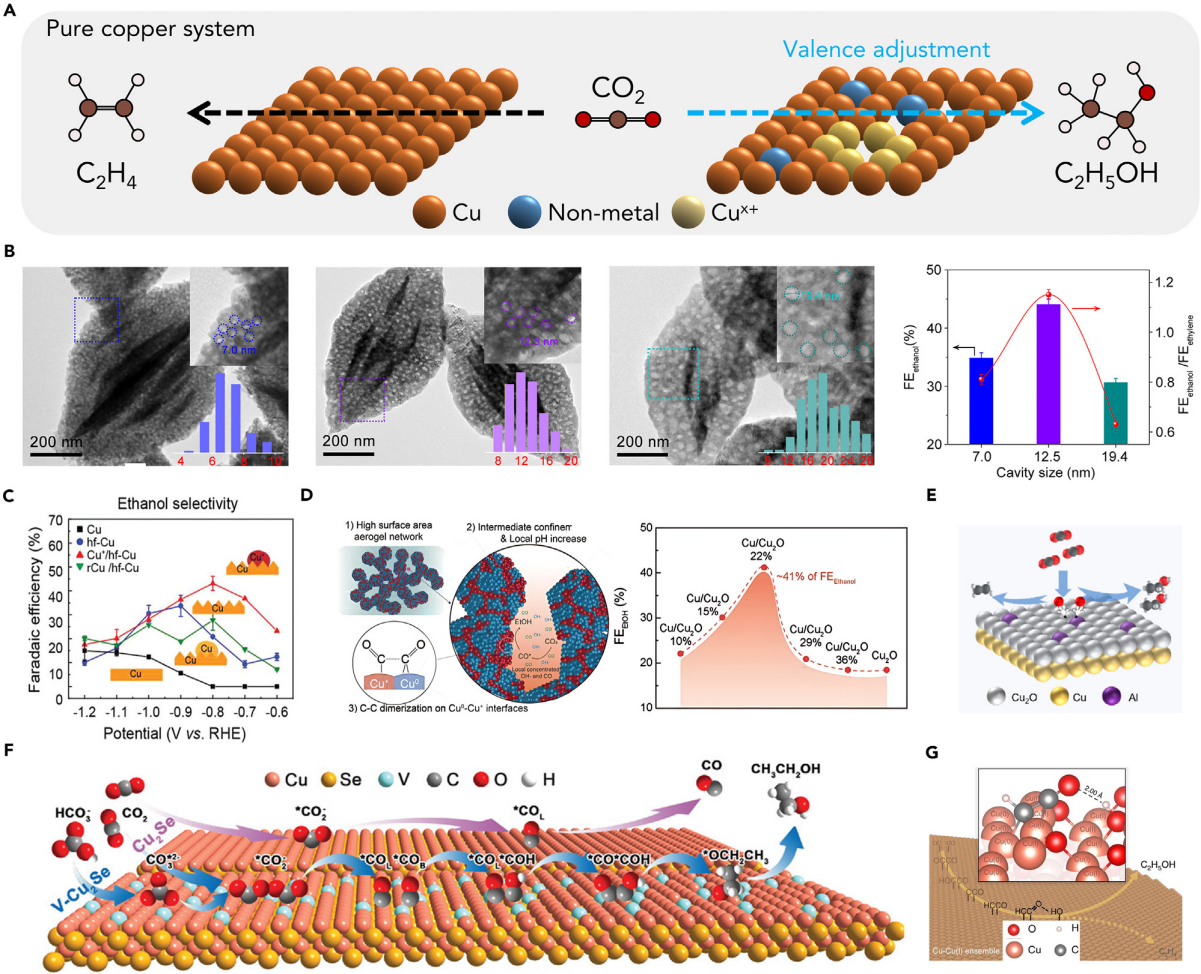
Oxidation state regulation of Cu catalysts (A) Schematic showing enhanced ethanol over ethylene production by optimizing the active site’s valence state. (B) TEM images of *p*-CuO-(7.0 nm, 12.5 nm, and 19.4 nm), the nanocavity structure is highlighted by dashed circles, and the inset illustrates size distributions of these nanocavities. Optimal FE ethanol and corresponding ethanol-to-ethylene ratio for the *p*-CuO-(7.0 nm), *p*-CuO-(12.5 nm), and *p*-CuO-(19.4 nm) catalysts.^71^ Copyright 2023 National Academy of Sciences. (C) Comparison of ethanol FE on polished Cu, hf-Cu, Cu^+^/hf-Cu, and rCu/hf-Cu.^72^ Copyright 2022, Wiley-VCH. (D) Proposed origin of the enhanced ethanol electrosynthesis performance on the Cu/Cu_2_O aerogel. Comparison of FE ethanol for Cu/Cu_2_O aerogels with the change in the Cu^+^ percentage.^73^ Copyright 2021, Wiley-VCH. (E) Schematic illustration of ethanol and ethylene formation paths on Al-Cu/Cu_2_O.^74^ Copyright 2023, American Chemical Society. (F) Schematic illustration for the catalytic mechanism for CO_2_RR to ethanol on V-doped Cu_2_Se nanotubes.^59^ Copyright 2022, Wiley-VCH. (G) The schematic representations of the determined factor for the selective ethanol synthesis.^75^ Copyright 2020, Springer Nature.

In the domain of CO_2_RR, the oxidation state of catalysts plays a pivotal role in determining their activity and selectivity.^56^^,^^57^^,^^58^^,^^59^^,^^60^^,^^61^ This is primarily due to the inherent ability of the oxidation state to influence key factors in the CO_2_RR process, which can be influenced by the oxidation states of catalysts from the following aspects: Firstly, the oxidation state affects CO_2_ activation, which is a critical step in initiating the electrochemical reduction of CO_2_. Cu-based materials demonstrate a unique synergy between Cu^+^ and Cu^0^, promoting CO_2_ activation.^62^^,^^63^^,^^64^ Cu^+^ sites can absorb H_2_O molecules, forming strong hydrogen bonds with CO_2_ and stabilizing activated molecules. They also help stabilize the transition state and final state of CO_2_ by diluting the negative charge. This insight guides the design of efficient CO_2_ electrocatalysts for sustainable conversion technologies. Secondly, it regulates the adsorption of intermediates, impacting the stability and reactivity of the reaction intermediates during the CO_2_ conversion process.^60^ Sargent and co-workers studied Cu-based catalysts with varying oxidation states (−0.1e to +0.3e).^65^ They observed that increasing the Cu oxidation state led to a consistent increase in ∗CO adsorption energy on the surface. This effect is due to changes in the electron state distribution and a higher overlap between C_2p_ and Cu_3d_ binding states when ∗CO adsorbs. Optimal binding strength with intermediates is crucial for enhancing catalyst performance and selectivity in CO_2_RR. Lastly, the oxidation state facilitates C−C coupling, a key step in the formation of higher-value C_2+_ products such as ethanol.^58^^,^^66^^,^^67^^,^^68^^,^^69^ Cu oxide (OD Cu) electrodes are typically obtained by growing Cu_2_O layers on the electrode surface from various Cu-based precursors (e.g., Cu foil, and Cu-based MOFs) at high temperatures and then reducing this oxide to form Cu^0^ sites. The presence of residual subsurface oxygen atoms enhances the CO binding energy to the catalyst by reducing the σ-repulsion, thereby favoring the kinetic mechanism for C−C bond formation due to increased ∗CO coverage on the catalyst.^70^ In summary, the oxidation state of catalysts holds substantial significance in CO_2_ electroreduction, as it directly influences the efficiency and selectivity of the process (Figure 5A). A deeper understanding of these influences can guide the rational design of more efficient and selective electrocatalysts for sustainable CO_2_ conversion to ethanol products.

Past studies indicate that the pathways for ethanol and ethylene formation diverge from a shared intermediate (∗CHCOH). The formation of ethylene occurs through the breaking of the C−O bond in ∗CHCOH, while ethanol production results from the protonation of ∗CHCOH.^17^^,^^32^^,^^76^ Given these findings, a potential strategy to enhance ethanol selectivity could be to increase the surface affinity for oxygenate intermediates. Given that the oxidized copper center can regulate the adsorption energy of intermediates and enhance C−C coupling, electrocatalytic materials designed around the metal oxidation state hold promise for improving the reduction of CO_2_ to ethanol. Therefore, Zhang et al. prepared a series of metal oxide nanoparticles with continuously changing pore sizes, the volcano-shaped relationship between ethanol selectivity and nanocavity size of porous CuO catalyst is found based on the well-controlled nanocavity size in the range of 0–20 nm (Figure 5B).^71^ They successfully showcased a highly selective and productive porous CuO catalyst, featuring an average nanocavity size of 12.5 nm for CO_2_RR-to-ethanol. As shown in Figure 5D, this catalyst exhibited an ethanol FE of 44.1 ± 1.0% and an ethanol-to-ethylene ratio of 1.2, at an ethanol partial current density of 501.0 ± 15.0 mA cm^−2^. The exceptional ethanol selectivity of the porous CuO catalyst is attributed to the increased coverage of surface-bounded hydroxyl species (∗OH), which results from the nanocavity size-dependent confinement effect. The theoretical calculations provide insights into the underlying mechanism, suggesting that the higher coverage of ∗OH preferentially facilitates the ∗CHCOH hydrogenation to ∗CHCHOH (ethanol pathway) by strengthening the noncovalent interaction. These findings highlight the significance of surface-bounded ∗OH species in directing the CO_2_RR toward ethanol production with high efficiency and selectivity. Jung et al. devised a novel electrocatalyst, denoted as (Cu^+^/hf-Cu), through a two-step process involving CVD graphene growth followed by a post oxidation step.^72^ In this process, insufficiently grown graphene grain boundary (gGB) defects acted as an oxidation-controlling mask, leading to the formation of Cu_2_O mesh patterns through gGB defects during post oxidation. Moreover, the Cu regions in the perfectly grown graphene regions exhibited high-facet surfaces, predominantly with (310) facets, and featured a high density of step-sites. The presence of both Cu^+^ sites and high-facet Cu on the electrocatalyst led to enhanced ∗CO adsorption, resulting in a rich ∗CO coverage that subsequently promoted ∗CO−∗CO reactions on the high-facet Cu surfaces. This catalytic mechanism significantly lowered the energy barrier for ethanol production, leading to a remarkable ethanol yield with 43% FE achieved at a low overpotential of −0.8 V vs. the RHE (Figure 5C). The (Cu^+^/hf-Cu) electrocatalyst exhibits promising potential for efficient and selective ethanol production in CO_2_ reduction reactions. Jung and his team have advanced their research by integrating diverse interfaces with porous structures, resulting in the creation of a porous Cu/Cu_2_O aerogel network (Figure 5D).^73^ This network serves as a platform for the electrochemical production of ethanol from CO_2_, demonstrating a remarkable ethanol current density. Examination through electron microscopy and electrochemical analysis reveals that the substantial enhancement in ethanol electrosynthesis can be ascribed to the abundance of Cu^0^/Cu^+^ interfaces. Furthermore, the confined porous aerogel network structure with its elevated surface area facilitates an increase in local pH, thereby contributing to the overall improved performance. These studies shed light on the pivotal role of oxidation states in electrocatalytic CO_2_ reduction to ethanol, paving the way for further research, albeit with a requirement for an improved understanding of reaction mechanisms and intermediate dynamics.

This section explores strategies to stabilize oxidation states in CO_2_ reduction to ethanol. The potential applied during the dioxide-reduction reaction is often much lower than the reduction potential of the Cu^δ+^ species, leading to the easy reduction of Cu^δ+^ species to Cu^0^ during the CO_2_RR process.^63^^,^^77^ Consequently, the selectivity and stability for ethanol over these Cu-based electrocatalysts remain low, and multiple liquid products other than ethanol are commonly obtained. This not only increases downstream separation expenses but also limits practical applications. To address this limitation, heteroatom doping emerges as a powerful strategy to finely tailor the active sites of the catalyst.^78^^,^^79^ By hybridizing energy levels between the dopant and the pristine catalyst, heteroatom doping can effectively control various intermediates and steer the reaction pathway toward the desired ethanol in CO_2_RR. Han et al., through a combination of DFT calculations and experimental validation, discovered that incorporating the Lewis acid metal Al can modulate the oxophilicity of Cu-based catalysts, thereby enhancing CO_2_ electroreduction to ethanol (Figure 5E). In flow-cell tests, Al-Cu/Cu_2_O demonstrated exceptional stability and achieved an impressive C_2+_ FE of 84.5%, with ethanol reaching 48.8% FE. *In situ* X-ray absorption spectroscopy (XAS) and emission spectroscopy (XES) analyses revealed that Al doping stabilizes Cu^+^ species during CO_2_ electrolysis. Further characterization, control experiments, and theoretical calculations have elucidated that Al doping facilitates the cleavage of the Cu−C bond of the selectively determining intermediate adsorbed at Cu sites, suppresses C−O bond cleavage, and stabilizes the oxygen-containing intermediate ∗OC_2_H_5_, thereby promoting the ethanol pathway. This strategy can be extended to other Lewis acid-site dopants, such as Ga and Mg.^74^ Sun et al. tackle the materials design challenge by introducing V-doped Cu_2_Se hierarchical nanotubes.^59^ The doping of V^4+^ ions into the Cu_2_Se lattice diversifies the active sites and prevents the reduction of Cu^+^ species to Cu^0^ during CO_2_RR (Figure 5F). The presence of multiple active sites enables the adsorption of bridge ∗CO_B_ species with a negative charge on V sites or Cu sites near the V sites, as well as the adsorption of linear ∗CO_L_ species with a positive charge on Cu sites away from the V sites. The high coverage of ∗CO_B_ facilitates the formation of ∗CO_H_, which subsequently couples with ∗CO_L_ to produce ethanol, as verified by *in situ* diffuse reflectance infrared fourier transform spectroscopy (DRIFTS) spectroscopy and DFT theoretical calculations. Remarkably, the optimal Cu_1.22_V_0.19_Se nanotubes catalyze CO_2_RR to ethanol with an FE of 67.3% and exhibit extraordinary long-term stability for 138 h in an H-cell. Additionally, they achieve a high partial current density of −207.9 mA cm^−2^ for ethanol production at −0.8 V in 1 M KOH in a flow cell. This study underscores the potential of V-doped Cu_2_Se hierarchical nanotubes as highly efficient and stable catalysts for selective CO_2_ electroreduction to ethanol. Conventional XAS and electron energy loss spectroscopy (EELS) techniques require minutes for spectrum acquisition, limiting their ability to capture dynamic changes during CO_2_RR. However, Lin et al. addressed this limitation by employing time-resolved XAS (TR-XAS) with a small X-ray incident angle.^75^ This innovative operando methodology allowed for seconds-resolved near-surface investigations of materials under CO_2_RR working conditions, enabling *in situ* characterization of catalytic surfaces. Using operando TR-XAS, the researchers tracked the chemical evolution of the material during CO_2_RR. Quantitative X-ray absorption near edge structure (XANES) and extended X-ray absorption fine structure (EXAFS) analyses revealed that the redox shuttle approach maintained a steady chemical composition, whereas conventional chronoamperometry significantly altered the material’s chemical nature. Remarkably, employing a potential switching approach led to the Cu^+^/Cu^0^ redox achieving a steady state of half-and-half during cathodic CO_2_RR electrolysis. This resulted in the asymmetric C_2_ product ethanol with high selectivity across a wide potential range. Theoretical computations suggested that a surface composed of Cu^0^-Cu^+^ ensembles could asymmetrically couple dual CO molecules, potentially enhancing the catalyst’s selectivity toward C_2_ products in CO_2_RR (Figure 5G). These strategies have demonstrated remarkable efficiency in stabilizing the valence state of Cu^δ+^ species, representing valuable insights for the development of oxidation-state catalysts. Furthermore, the understanding gained from these studies can serve as a valuable reference for the design and optimization of advanced catalysts tailored for specific applications, ranging from energy conversion and storage to environmental remediation and sustainable chemical synthesis.

## Alloy catalysts

**Figure 6:**
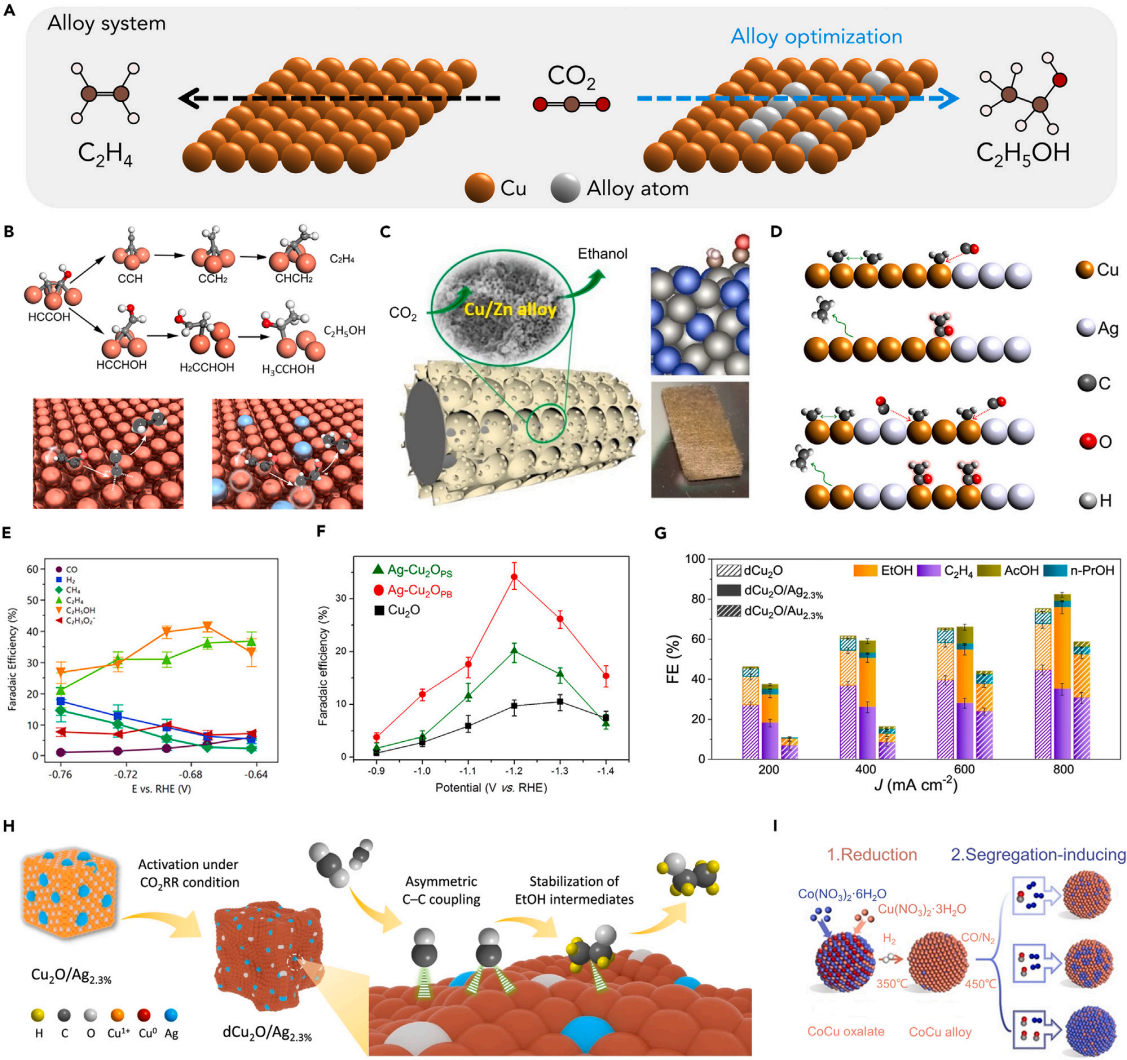
Alloy catalysts (A) Schematic showing enhanced ethanol over ethylene production by alloying treatment. (B) Reaction paths for ethylene vs. ethanol on a Cu (111) surface. Binding illustration for Cu and Ag/Cu catalyst to produce ethylene and ethanol, respectively.^87^ Copyright 2019, American Chemical Society. (C) Schematic diagram of the generation of ethanol from CuZn alloy with porous structure.^91^ Copyright 2020, Elsevier. (D) Hypothetical CO-insertion mechanism scheme indicating the transfer of CO from one metal site that weakly binds CO (Ag) to another site that binds residual C_1_ intermediate species (Cu) in the case of Ag-Cu_2_O_PS_ and Ag-Cu_2_O_PB_.^92^ Copyright 2017, American Chemical Society. (E) Ag_0.14_/Cu_0.86_ catalyst FE toward the major CO_2_ reduction products.^87^ Copyright 2019, American Chemical Society. (F) The FE of ethanol for Cu_2_O, Ag-Cu_2_O_PS,_ and Ag-Cu_2_O_PB_.^92^ Copyright 2017, American Chemical Society. (G) FE value of C_2+_ products for dCu_2_O, dCu_2_O/Au_2.3%_ and dCu_2_O/Ag_2.3%_ under selected current density.^20^ Copyright 2022, Springer Nature. (H) Schematic for boosted ethanol generation over dCu_2_O/Ag_2.3%_. Yellow-color, gray, white, orange, red, and azure spheres in the model represent H, C, O, Cu^+^, Cu^0^, and Ag atoms, respectively.^20^ Copyright 2022, Springer Nature. (I) The synthesis methodology for CoCu alloys with different Co surface segregation degrees: low, moderate, and high.^93^ Copyright 2022, Wiley-VCH.

CO_2_RR tends to develop C_2_ high-value products and harsh reaction conditions. The uniform surface with a homoatomic center is difficult to provide a high-energy catalytic center for multi-carbon products and keep good durability under acidic and alkaline conditions.^80^^,^^81^ Recently, alloy catalysts have been reported to overcome the above obstacles.^82^ Li et al. discovered that when two metals interact, it directly affects the d-band center of each metal, a critical factor in determining adsorption and desorption energies.^83^ In CO_2_ reduction, excessively low adsorption energy impedes CO_2_ activation, while overly strong adsorption energy inhibits product desorption. Therefore, designing alloy catalysts with a rational structure is essential for effective CO_2_ reduction.^81^ Metal alloying has the following advantages: (1) in alloy catalysts, the surface potential and d-band center can be easily tuned by different compositions, which affect the adsorption and activation behaviors of reactants and intermediates.^84^^,^^85^^,^^86^ The first step of CO_2_ reduction is the adsorption of CO_2_ molecules. The interaction of two metals usually provides sufficient catalytic sites to achieve high adsorption energy. (2) alloy catalysts provide multi-metal active centers that are conducive to the construction of asymmetric active centers or provide an asymmetric reaction environment, which is beneficial to the formation of asymmetric products.^20^^,^^87^ Considering that the production of ethanol in CO_2_ reduction reactions requires the initial coupling of two adjacent ∗CO intermediates on Cu atoms, it necessitates a high local concentration of CO and a substantial coverage of ∗CO intermediates on the catalyst surface.^88^^,^^89^ Therefore, CO-generating metals such as Ag, Au, or Zn are often selected as promoter elements in M-Cu bimetallic systems, a concept known as sequential catalysis (Figure 6A). The incorporation of these promoter elements has been shown to significantly enhance the selectivity toward ethanol formation.^90^

In recent years, remarkable progress has been made in electrochemical CO_2_ reduction, especially in increasing C_2+_ productivity. However, achieving high selectivity for ethanol remains challenging compared to ethylene and other products. To address this issue, Li et al. introduced diverse binding sites to a Cu catalyst, destabilizing ethylene intermediates and promoting ethanol production (Figure 6B). They synthesized electrocatalysts by co-sputtering Ag and Cu on a polytetrafluoroethylene (PTFE) substrate, achieving a record FE of 41% for ethanol toward ethanol at 250 mA cm^−2^ and −0.67 V vs. RHE (Figure 6E). DFT calculations validated the improved ethanol path and reduced ethylene pathway. The newly developed catalysts display a significantly broader *in situ* Raman spectrum in the ∗CO stretching region compared to pure Cu controls.^87^ This observation can be attributed to the diverse binding configurations. The physical picture of multisite binding explains the improved ethanol production in bimetallic catalysts and provides a framework for designing multi-metallic catalysts to control reaction paths in CO_2_ reductions toward ethanol products. Besides, Lee et al. investigated Ag-incorporated Cu_2_O materials, phase-separated Ag-Cu_2_O_PS_, and phase-blended (PB) Ag-Cu_2_O_PB_.^92^ Ethanol selectivity significantly improved with Ag incorporation, reaching 20.1% for Ag-Cu_2_O_PS_ and 34.15% for Ag-Cu_2_O_PB_, compared to 10.5% for Cu_2_O-Cu without Ag (Figure 6F). Surface characterizations showed stable Cu-to-Ag ratios after electrode reduction, with Ag-Cu_2_O_PB_ having higher Ag content. However, Ag content alone couldn’t fully explain the different ethanol product FE. The atomic arrangement also influenced selectivity, with Ag-Cu_2_O_PB_’s PB pattern favoring closer Ag-Cu proximity, facilitating CO insertion and promoting ethanol production (Figure 6D). This study highlights co-catalyst nature and the significance of atomic arrangement in enhancing ethanol selectivity during CO_2_ reduction. CuAg alloys may not universally favor ethanol production over ethylene. Hoang et al. demonstrated that a CuAg catalyst prepared via co-electrodeposition on carbon paper exhibited higher selectivity toward ethylene than ethanol.^28^ This suggests that the choice of preparation method for the alloys can be a critical factor influencing the selectivity between ethylene and ethanol.

Similar to Ag, Zn is a well-known catalyst for CO_2_ reduction to CO^94^; therefore Ren et al. conducted a study on Cu-based alloy catalysts with different quantities of Zn dopants (Cu, Cu_10_Zn, Cu_4_Zn, and Cu_2_Zn) for electrocatalytic reduction.^91^^,^^95^ The addition of Zn played a crucial role in continuously producing an *in situ* source of mobile CO during the electrolysis. This mobile CO diffused to neighboring Cu sites and reacted with other C_1_ intermediates (∗CO or ∗CH_2_). The researchers found that the ratio of FE_ethanol_/FE_ethylene_ could be finely tuned by a factor of up to around 12.5. Among the catalysts, Cu_4_Zn showed the highest FE_ethanol_ of 29.1%, while bare Cu had the highest FE_ethanol_ of 11.3%. Interestingly, the FEs of CO significantly decreased simultaneously with the increase in FEs of ethanol, indicating that gaseous CO formed from the Zn sites was further reduced to ethanol. Zhao et al. developed porous bimetallic Cu/Zn catalysts using a wet-chemical synthesis method, allowing them to tune the alloy composition by varying metal precursor ratios (Figure 6C).^91^ These catalysts demonstrated catalytic synergies of Cu and Zn, promoting CO_2_ electroreduction to liquid ethanol products. DFT calculations revealed that electron-rich Cu in the bimetallic catalysts enhanced CO_2_ adsorption while suppressing H_2_ adsorption. Zn doping in Cu promoted the formation of ∗CO intermediates, weakly covering the Cu surface, increasing the energy barrier for proton reduction, and improving the overall FE for CO_2_ reduction. The hierarchical porosity of the bimetallic catalysts extended the retention time of intermediates, facilitating the deep reduction of CO_2_ to form liquid products. Notably, the porous Cu_5_Zn_8_ catalyst exhibited an impressive 46.6% FE for ethanol production at −0.8 V during steady-state electrolysis for 11 h. This study highlights the potential of porous Cu/Zn alloy catalysts for efficient and selective CO_2_ electroreduction to valuable liquid fuels.

In the preceding section, we extensively explored the crucial role played by the oxidation state in influencing the conversion of CO_2_ into ethanol, a preferred outcome over ethylene. Wang et al. ingeniously devised a novel and promising approach. By synergizing alloy catalysts with metal oxidation-state catalysts, they aimed to create a powerful combination that could enhance the selectivity for the production of ethanol during the electrochemical reduction of CO_2_.^20^ Specifically, the study focused on modifying cubic Cu_2_O with Ag and exploring CuAg bimetallics (dCu_2_O/Ag) with controlled morphology, phase, and composition for efficient CO_2_RR to ethanol at high current densities. Unlike Cu_2_O and Au-modified Cu_2_O derivatives that favor CO_2_ conversion to C_2_H_4_ and CO, respectively, the optimized dCu_2_O/Ag_2.3%_ catalyst exhibited a unique asymmetric C−C coupling, enhancing ethanol production under high current density conditions. In the flow cell (Figure 6G), the optimized dCu_2_O/Ag_2.3%_ catalyst demonstrated impressive results, with an FE of 40.8% and an energy efficiency for ethanol of 22.3%, along with a remarkable ethanol partial current density of 326.4 mA cm^−2^ at −0.89 V vs. RHE (with an 85% iR correction). *In situ* attenuated total reflection infrared absorption spectroscopy (ATR-IRAS) confirmed that the improved ethanol selectivity resulted from moderate surface coordination and optimal oxidation state of the Cu sites, facilitating mixed ∗CO_bridge_ and ∗CO_atop_ configurations that stabilized the ethanol intermediates (Figure 6H). These groundbreaking insights into the CO_2_-to-ethanol electroreduction mechanism offer a distinct departure from conventional CO-tandem catalysis. In a groundbreaking study, Liu et al. introduced a novel perspective on alloying elements beyond the conventional choices of Ag, Zn, and Au. Utilizing a theoretical surface phase diagram derived from extensive global searching, they revealed the potential of Co as a segregating element on the CoCu surface (Figure 6I).^93^ This segregation led to a significant increase in CO coverage, profoundly influencing catalytic performance through surface segregation and coverage effects. Computational predictions highlighted the catalytic implications of Co segregation, resulting in enhanced C−O scission of ∗CH_2_O and competent C−C coupling, ultimately yielding a highly desirable high ethanol selectivity over the CoCu surface. To validate their theoretical insights, Liu et al. executed a two-step reduction-induction procedure, successfully achieving remarkable >60% ethanol selectivity over the moderately CO-induced CoCu catalyst. Even in the face of potential surface oxidation and restructuring during the reaction, the catalyst’s performance remained remarkably robust. The ability to fine-tune alloy systems with segregation tendencies under specific conditions represents a pivotal step toward achieving efficient and selective catalytic processes, particularly in the realm of CO_2_ electroreduction to value-added products like ethanol. The use of alloys for the reduction of CO_2_ to ethanol presents tremendous advantages and holds enormous promise for sustainable energy conversion.

## Tandem catalysts

**Figure 7:**
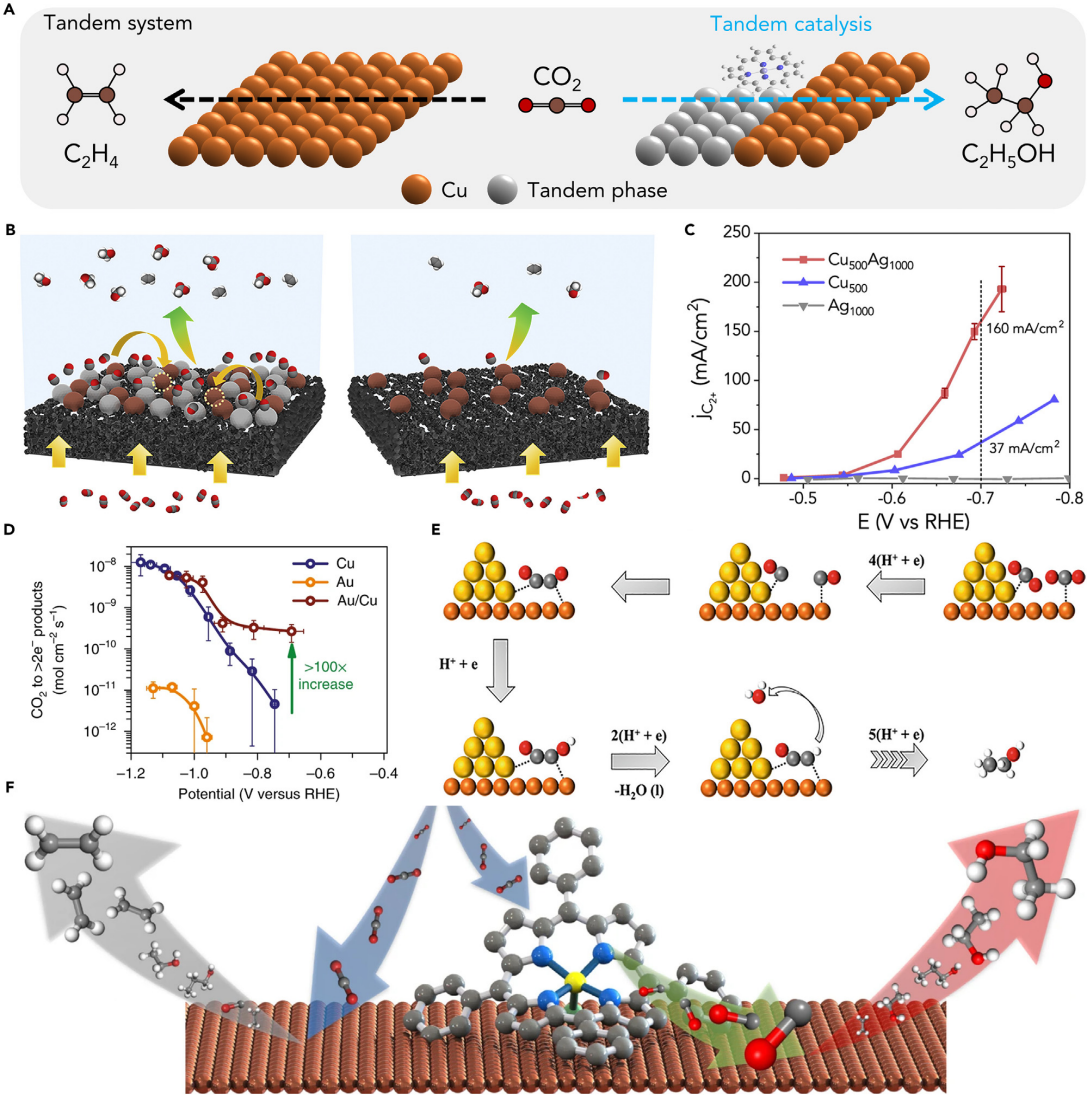
Tandem catalysts (A) Schematic showing enhanced ethanol over ethylene production by tandem catalysis. (B) Schematic illustration of Cu-Ag nanoparticle tandem catalyst and Cu nanoparticle catalyst of the reduction of CO_2_ to ethanol.^104^ Copyright 2020, Cell Press. (C) The partial current density of C_2+_ product of different catalysts.^104^ Copyright 2021, Cell Press. (D) The rate of CO_2_ reduction to >2e^−^ products of different catalysts.^31^ Copyright 2023 National Academy of Sciences. (E) Schematic illustration of Cu/Au tandem catalyst of the reduction of CO_2_ to ethanol.^21^ Copyright 2023 National Academy of Sciences. (F) Schematic illustration of FeTPP[Cl] on a Cu tandem catalyst and pure Cu electrode of the reduction of CO_2_ to different C_2+_ product.^105^ Copyright 2020, Springer Nature.

Due to the chemical inertness of CO_2_, it is often difficult to convert it directly into ethanol at pure Cu catalyst. Despite this, the CO_2_ can firstly convert to ∗CO at extra component, and then the ∗CO can be further used to produce C_2_ product at Cu sites (Figure 7A). This special catalyst that consisting two different components was called tandem catalysts.^96^^,^^97^^,^^98^^,^^99^^,^^100^^,^^101^^,^^102^^,^^103^ In CO_2_RR field, many precious metal materials and SACs have been studied for electrochemical catalyzing CO_2_ to CO and show excellent selectivity. Therefore, these catalysts are preferred as the first part of a tandem system. Chen et al. physically mixed Cu nanoparticles with Ag nanoparticles and loaded them on the GDL (Figures 7B and 7C).^104^ Since Ag can efficiently generate CO, and Ag and Cu are in close contact, a CO-enriched environment is formed near Cu, which is favorable to the conversion of CO into C_2+_ product by Cu. Therefore, the activity to generate C_2+_ products was significantly increased to J_C2+_ = 160 mA cm^−2^ (about four times) compared with Cu. This unique and simple method provides a new idea for future catalysts design. If a local high-concentration CO environment can be effectively constructed near the Cu sites, it may be beneficial to the production of ethanol. Based on this idea, researchers began to consider coupling catalysts that can efficiently produce CO with Cu to obtain tandem catalysts with ideal performance. Carlos et al. deposited gold nanoparticles on a fairly flat polycrystalline Cu foil by physical vapor deposition to obtain Au/Cu tandem catalyst (Figure 7D).^31^ They ruled out the influence of other aspects such as the Au/Cu alloy interface through experiments and confirmed that the performance improvement comes from the tandem mechanism, where Au and Cu are in close proximity and have complementary surface chemistry. Remarkably, at low overpotentials, the rate of CO_2_ reduction to >2e^−^ products is more than 100 times higher on Au/Cu than on Cu. Kuang et al. prepare bimetallic electrocatalysts based on Cu/Au heterojunctions with an FE toward ethanol of 60% at currents in excess of 500 mA cm^−2^. By comparing with CuAu alloy, Cu nanoparticles, Au nanoparticles and Cu nanoparticles and Au nanoparticles physically mixed catalyst, using *in situ* ATR-IR confirmed that this heterojunction is beneficial to the formation of OCCOH∗, which in turn can improve the selectivity of ethanol. Based on DFT results, the adsorption sites of the two C atoms of ∗CO-∗CO are on Cu and Au, respectively, which leads to the asymmetric hydrogenation of ∗CO on the Cu site, which in turn promotes the formation of key intermediate (Figure 7E).^21^ Besides noble metals, SACs are also an alternative suitable catalyst for CO production. Li et al. immobilized the FeTPP[Cl] on a Cu electrode sputtered on a hydrophobic porous PTFE substrate to realize CO_2_-to-ethanol conversion with an FE of 41% at a partial current density of 124 mA cm^−2^ (Figure 7F).^105^ Notably, the DFT result confirm that the reaction energy decreased more for the formation of ∗CHCHOH (ethanol path) compared with that of ∗CCH (ethylene path). So, they show for the first time that increasing ∗CO content can modulate the selectivity of ethylene to ethanol. This conclusion was also confirmed by varying the loading of FeTPP[Cl] and using other TPP catalysts. In summary, the design ideas of the tandem catalysts are the same. The researchers hope to promote the C−C coupling process by constructing a high-concentration ∗CO environment around the Cu catalyst, so as to achieve efficient ethanol production. However, it should be emphasized that just promoting C−C coupling still cannot achieve high selectivity for ethanol, because this process may also promote the formation of ethylene, so other means are needed to inhibit the detachment of hydroxyl groups.^106^^,^^107^ But in any case, under normal circumstances, C−C coupling is the rate-determining step of C_2+_ products, and usually lowering the energy barrier of this reaction is beneficial to the formation of C_2+_ products like ethanol.

## Composite catalysts

**Figure 8:**
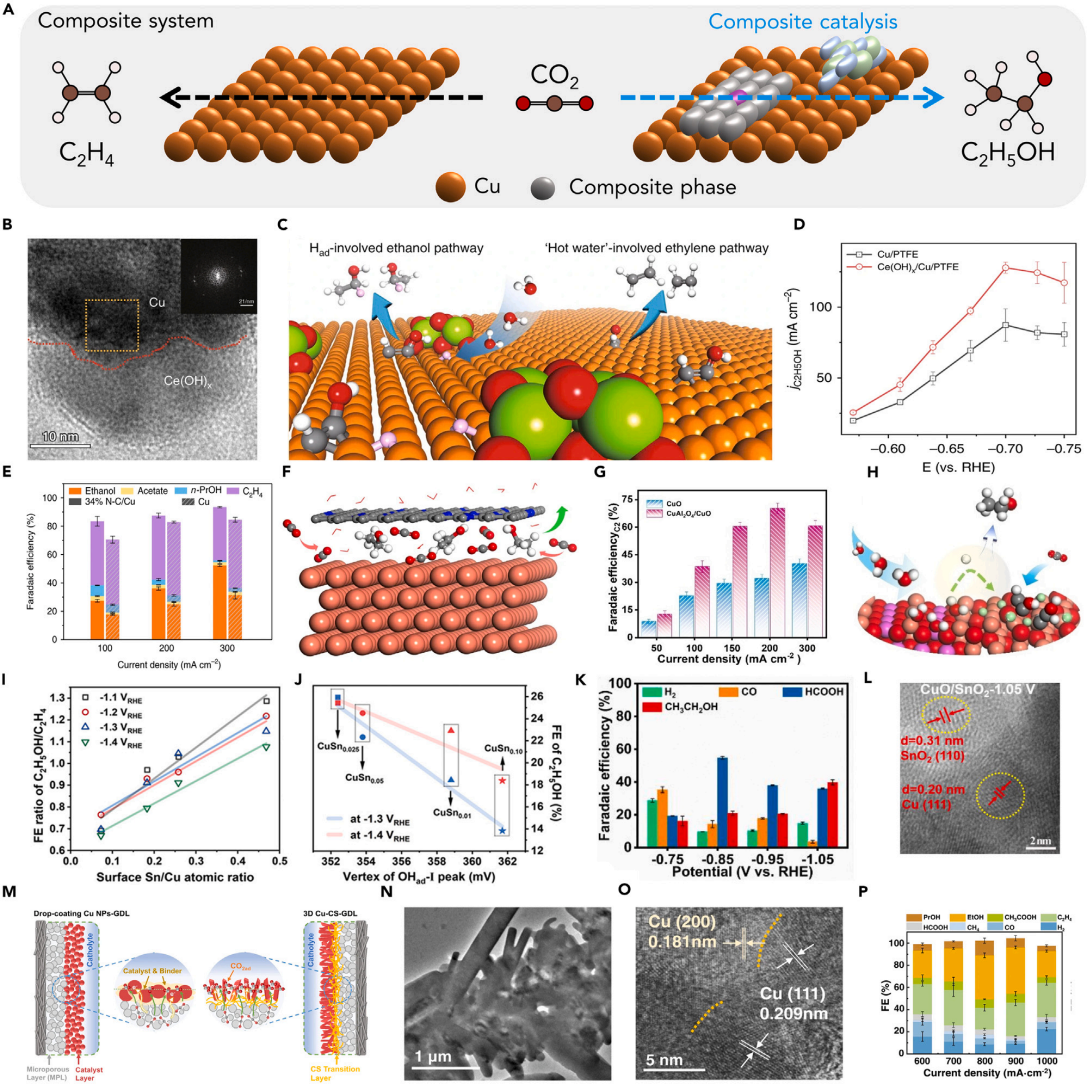
Composite catalysts (A) Schematic showing enhanced ethanol over ethylene production by composite catalysis. (B) The high-resolution TEM image of Ce(OH)_x_/Cu/PTFE catalyst.^109^ Copyright 2019, Springer Nature. (C) Schematic illustration of Ce(OH)_x_/Cu/PTFE catalyst CO_2_ to ethanol.^109^ Copyright 2019, Springer Nature. (D) The j_ethanol_ comparison between Ce(OH)_x_/Cu/PTFE and Cu/PTFE.^109^ Copyright 2019, Springer Nature. (E) The FE of NC−Cu and Cu catalysts.^32^ Copyright 2020, Springer Nature. (F) Schematic illustration of NC−Cu reduced CO_2_ to ethanol.^32^ Copyright 2020, Springer Nature. (G) The FE of CuAl_2_O_4_/CuO and CuO catalysts.^110^ Copyright 2023, Wiley-VCH. (H) Schematic illustration of CuAl_2_O_4_/CuO concentrated ∗H on the surface to facilitate ethanol production.^110^ Copyright 2023, Wiley-VCH. (I) The FE ratio of C_2_H_4_ and ethanol between different surface Sn/Cu atomic ratio catalysts.^85^ Copyright 2023, Wiley-VCH. (J) Linear relationship between FE_ethanol_ and OH^−^ peak position.^85^ Copyright 2023, Wiley-VCH. (K) The FE of different products of CuO/SnO_2_ catalyst at different potential.^61^ Copyright 2023, Wiley-VCH. (L) The high-resolution TEM image of CuO/SnO_2_ after reconstructed to Cu/SnO_2-x_ at −1.05 V (vs. RHE).^61^ Copyright 2023, Wiley-VCH. (M) Schematic illustration of the drop-coating Cu NPs-GDL and 3D Cu-CS-GDL micro structure.^111^ Copyright 2023, Springer Nature. (N) The low-resolution TEM image of the hexagonal prismatic Cu microrods (3D Cu-CS-GDL).^111^ Copyright 2020, Springer Nature. (O) The high-resolution TEM image of the hexagonal prismatic Cu microrods (3D Cu-CS-GDL).^111^ Copyright 2020, Springer Nature. (P) The FE of different product of 3D Cu-CS-GDL catalyst at different current density.^111^ Copyright 2020, Springer Nature.

According to the current understanding of most studies, it is difficult for Cu catalysts to directly convert CO_2_ into ethanol. The most important reason for this is the inability to control the dissociation of hydroxyl group of key intermediate products.^74^ Therefore, the multi-carbon products produced by most Cu catalysts are dominated by ethylene (Figure 8A). Nevertheless, high ethanol selectivity can be achieved if some other components are composited on the Cu catalyst, and these components can limit the C−O bond broken.^108^ Luo et al. deposit Ce(OH)_x_ on Cu/PTFE electrode using the electrochemical deposition method to get the Ce(OH)_x_/Cu/PTFE catalyst (Figures 8B–8D).^109^ The introduction of Ce(OH)_x_ can regulate the surface coverage of ∗H, thereby attacking the ∗HCCOH, forming ∗HCCHOH, the key intermediate toward ethanol, promoting ethanol production. In addition, due to the increase of the surface ∗H coverage, the hydrogen bond of H_2_O to the hydroxyl group is weakened, thereby inhibiting the breaking of the C−O bond. The Ce(OH)_x_/Cu/PTFE can reach FE of 43% for ethanol at an operating current density of 300 mA cm^−2^. Wang et al. sputtering a layer of N−C on the surface of Cu/PTFE which can promote C−C coupling and suppresses the breaking of the C−O bond in HOCCH∗, thereby promoting ethanol selectivity in CO_2_RR (Figures 8E and 8F).^32^ The catalyst delivers an ethanol FE of 52 ± 1% with a conversion rate of 156 ± 3 mA cm^−2^. Notably, the authors found through DFT that the N element in the surface capping layer is crucial to promote C−C coupling, it is beneficial to electron transfer to adsorbed ∗CO on Cu.

In addition to the strategies mentioned above to suppress C−O bond breakage, ethanol selectivity can also be improved by changing the oxophilicity and hydrophobicity of the catalyst.^34^ If the surface of the catalyst is hydrophilic, the surface is easily covered with a large amount of ∗H produced by the decomposition of H_2_O, and these ∗H are easily combined with the generated ∗CO intermediate products to protonate them, and then form ∗CHO, ∗CH_3_, and other intermediate products. When these intermediate products are further coupled, the final product will be ethanol. However, it should also be noted that the regulation of hydrophilicity needs to be very careful. Because if the hydrophilicity is too strong, the hydrogen evolution reaction (HER) activity of the catalyst may be significantly improved, which is not conducive to CO_2_RR. Zhang et al. prepared the CuAl_2_O_4_/CuO via calcining CuAl-layered double hydroxide (CuAl-LDHs) precursor at 800°C in a pure oxygen atmosphere.^110^ The authors demonstrated that CuAl_2_O_4_ can promote the decomposition of H_2_O by kinetic isotopic effect, *in situ* electrochemical impedance spectroscopy (EIS), and ∗H trapping experiment, which in turn is beneficial to the increase of ∗H coverage. In addition, DFT calculations also show that ∗H preferentially adsorbs at the O sites and the ∗H spillover has a lower energy barrier on CuAl_2_O_4_, which suggests that increased ∗H coverage facilitates the migration of ∗H to the hydrogenation of subsequent intermediates, thereby boosting ethanol formation. The catalyst delivered an FE of 41% for ethanol at current density of 200 mA cm^−2^ and exhibited a continuous 150 h durability in a flow cell (Figure 8G). Regulating the oxophilicity of the catalyst is to prevent the detachment of O, so as to ensure the stable existence of oxygen-containing functional groups (Figure 8H). Li et al. synthesized a series of dendrite Cu-Sn bimetallic catalysts with different Sn content. Due to the introduction of Sn, the oxophilicity of CuSn_x_ is enhanced compared to Cu (Figure 8I).^85^ They used hydroxide adsorption experiments to correlate the oxophilicity with the Sn content, thus confirming that the higher the Sn content, the higher the oxophilicity (Figure 8J). And found that when the oxophilicity is controlled in a suitable range (CuSn_0.025_), ethanol has the best selectivity (FE_ethanol_ = 25.93% on CuSn_0.025_ at −1.4 V with a large partial current density of 15.05 mA cm^−2^). In addition, this work also introduced other metals with oxophilicity (Pb, Ag), which also promoted the production of ethanol, further proving their conclusion.

Except the conventional modification methods of catalysts, the use of catalysts to undergo structural evolution under reduction potentials can also promote the production of ethanol. However, the change in performance brought about by the evolution of the structure is often only for a certain catalyst, and the root cause of the deeper structural change is still unclear, so more in-depth research is still needed. Wang et al. explored the evolution of CuO/SnO_2_ catalyst under reducing conditions, and it was found that the catalysts evolved at different potentials had different selectivity (Figure 8K).^61^ At −0.85 V (vs. RHE), the CuO/SnO_2_ evolves to Cu_2_O/SnO_2_ with high selectivity to HCOOH (FE of 54.81%). But the difference is reconstructed to Cu/SnO_2-x_ at −1.05 V (vs. RHE) with significantly improved FE to ethanol of 39.8%. Due to the special interface structure of Cu/SnO_2-x_ produced by reconstruction and observed by transmission electron microscopy (TEM) (Figure 8L), the special absorption configurations of ∗COOH and ∗CHOCO via both C and O can interact, favors the ∗CO formation and lowers the energy barrier for C−C coupling, and then promoting the production of ethanol, which is detected by *in situ* Raman test and DFT calculation. Although the interface constructed in this work has special effects, it is actually difficult to construct such an interface through other types of catalysts. Therefore, it is difficult to have universal applicability for the electrochemical conversion of CO_2_ to ethanol. Bi et al. prepare 3D Cu-chitosan-GDL electrode for efficient ethanol production.^111^ Due to this special three-dimensional structure, chitosan can be used as a transition layer to connect the catalyst and the electrode, thereby promoting the structural change of the electrode to form a three-dimensional Cu film (Figure 8M). The resulting hexagonal prismatic Cu microrods have excellent ethanol catalytic performance, which shows alcohols selectivity is 51.4% with a partial current density of 462.6 mA cm^−2^ (Figure 8P). The special role of chitosan can not only change the catalyst structure to generate abundant (200)/(111) grain boundaries (Figures 8N and 8O), but also reduce or eliminate the interfacial contacting resistance between the catalyst and substrate, which is beneficial to the reaction. This design idea is quite interesting, because a special chitosan is used to modify the electrode not only produce special grain boundaries to facilitate ethanol production, but also reduce the charge transfer resistance, which can be said to kill two birds with one stone. In general, the deep-seated mechanism of ethanol selectivity improvement caused by structural changes is worthy of further exploration and in-depth understanding.

## Non-copper catalysts

**Figure 9:**
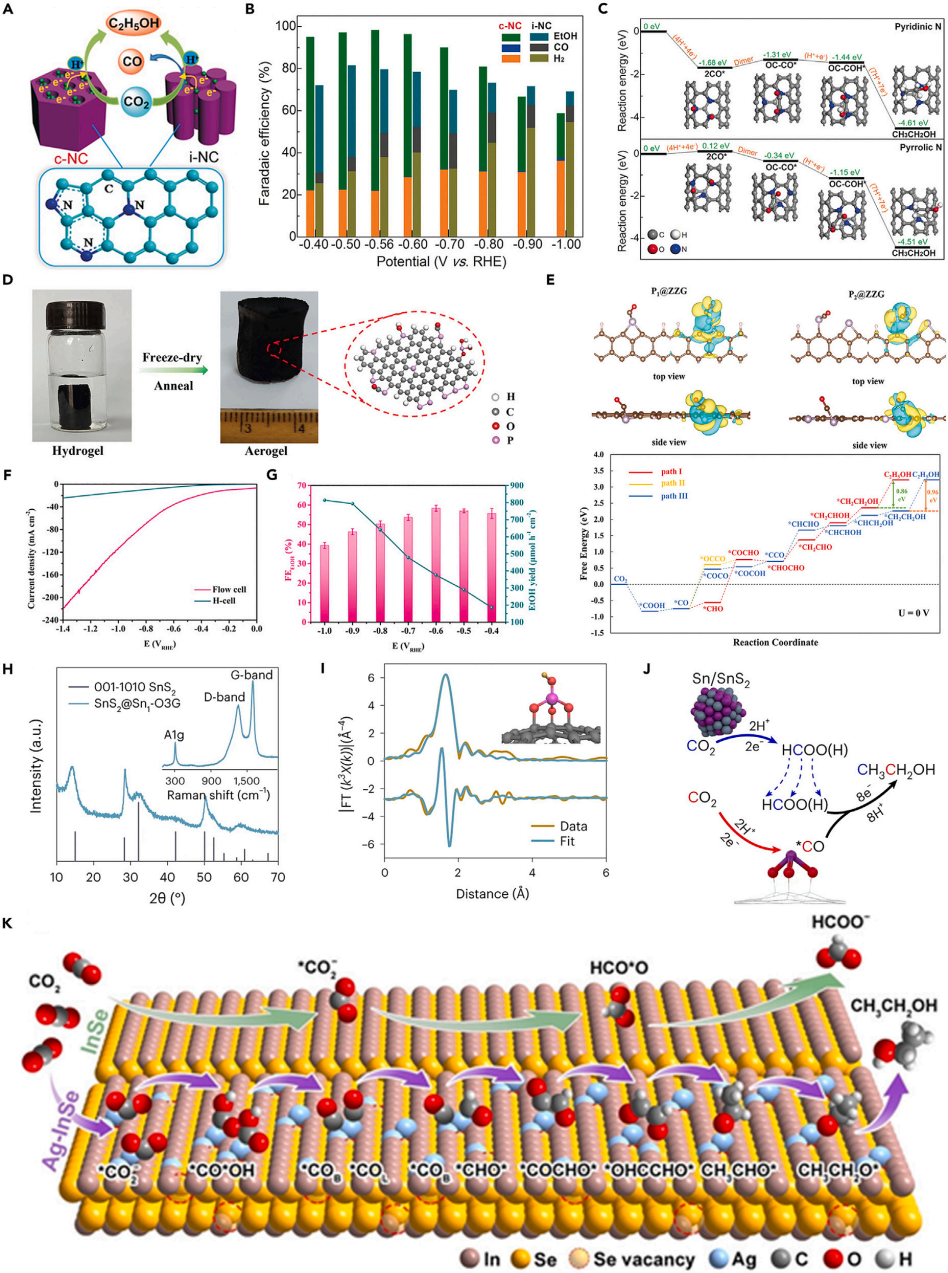
Non-copper catalysts (A) Schematic illustration of c-NC converts CO_2_ to ethanol.^113^ Copyright 2017, Wiley-VCH. (B) The FE of c-NC and i-NC under different potential.^113^ Copyright 2017, Wiley-VCH. (C) The reaction energy diagram of different active N sites.^113^ Copyright 2017, Wiley-VCH. (D) Schematic illustration of the synthesis process of PGAs.^114^ Copyright 2022, Wiley-VCH. (E) The top and side view of P_1_@ZZG and P_2_@ZZG and free energy diagram of reaction coordinate steps.^114^ Copyright 2022, Wiley-VCH. (F) LSV curves of PGA-2 tested in H-cell and flow cell.^114^ Copyright 2022, Wiley-VCH. (G) Ethanol FE and yield on PGA-2 in flow cell.^114^ Copyright 2022, Wiley-VCH. (H) XRD pattern of the SnS_2_/Sn_1_-O_3_G with the Raman spectrum provided in the inset.^115^ Copyright 2023, Springer Nature. (I) First shell fitting of the Fourier transformation of the EXAFS spectrum of Sn_1_-O_3_G.^115^ Copyright 2023, Springer Nature. (J) Schematic illustration showing the cascade reaction during CO_2_ reduction to ethanol over SnS_2_/Sn_1_-O_3_G (gray: S, red: O, yellow: H and purple: Sn).^115^ Copyright 2023, Copyright 2023, Springer Nature. (K) Schematic illustration for the catalytic mechanism of ethanol production in CO_2_RR on Ag^+^-doped InSe. In: orange; Se: yellow; Ag: blue; C: gray; H: white; O: red.^116^ Copyright 2023, Wiley-VCH.

In addition to the Cu-based catalysts, there are currently some non-copper catalysts that can produce ethanol, including carbon materials with heteroatom doping and porous structure.^112^ These doped heteroatoms such as N and P can serve as functional active sites to promote CO_2_ conversion. Song et al. synthesized mesoporous N-doped carbons tailored with highly uniform cylindrical channel structures (named as c-NC) via the self-assembly approach (Figure 9A).^113^ Both pyridinic and pyrrolic N sites are the active sites for generating ∗CO. Besides, due to the special cylindrical channel and high electron density of surface existing in the c-NC, the ∗CO intermediates can be stabilized. The C−C bond formation is also favored at pyridinic N sites compared with pyrrolic N, whose reaction energy is −1.31 eV (Figure 9C). Certainly, the electron-rich surface can also promote the proton-electron transfer process, thus favoring the formation of ∗OC−COH. The c-NC can reach FE_ethanol_ = 77% at the low potential of 0.56 V (vs. RHE), which is one of the highest selectivity until now (Figure 9B). Yang et al. reported P-doped graphene aerogels (PGAs) catalyst shows ethanol FE = 48.7% at −0.8 V (vs. RHE) (Figures 9D and 9E).^114^ This material was prepared by a hydrothermal reduction of dispersed graphene oxide aqueous solution with phosphoric acid, and then freeze-drying and subjected to thermal treatment (Figure 9D). The author confirmed that the P connected with two carbon atoms at the graphene zigzag edge is the real active site, which can chemically bind ∗CO with negative binding energy for further C−C coupling (Figure 9E). Also, the DFT results show that the energy barrier for ∗CH_2_CHOH→ ∗CH_2_CH (1.37 eV) is much higher than that of ∗CH_2_CHOH→ ∗CH_2_CH_2_OH (0.45 eV), confirming that ethanol is the more possible C_2_ product.

There are other metal elements that also have the ability to produce ethanol, such as Sn, Co, etc. These metal sites are extremely selective in producing ethanol in a specific catalyst system. For example, Liu et al. synthesized a special tin-based catalyst SnS_2_/Sn_1_-O_3_G.^115^ Through X-ray diffraction (XRD), EXAFS, isotope labeling, and reactant control experiments (Figures 9H and 9I), it can be seen that this catalyst contains two kinds of active sites, one of which can produce formic acid is Sn/SnS_2_ site, while the other is the SnO_3_(OH)G site that can produce ∗CO and undergo C−C coupling. It should be noted that the carbon support used in this article does not affect the selectivity and only plays a conductive role. Similar to previous work, the author found that Sn/SnS_2_ nanosheet only have the ability to produce HCOOH, but because the catalyst has special SnO_3_(OH)G sites, HCOO (H) can further couple with ∗CO to generate ethanol (Figure 9J). The FE of the SnS_2_/Sn_1_-O_3_G for ethanol product can reach an astonishing 82.5%, making it the most selective non-copper-based catalyst currently. However, this catalyst can only measure such high performance in an H-type cell. When using a flow cell, the FE_ethanol_ drops to 65%, so this catalyst is difficult to use at high current densities, and its activity still needs to be improved. Xiong et al. incorporated Ag into InSe and adjusted the doping content to obtain the Ag_0.015_In_0.985_Se_0.734_ catalyst.^116^ In this work, the author tested the performance of the catalyst in the Membrane electrode assemblie (MEA). The FE_ethanol_ can reach 68.7%, and the partial current density reaches 186.6 mA cm^−2^ on the cathode with a full-cell ethanol energy efficiency of 26.1% at 3.0 V. The reason for the excellent performance of this catalyst is the introduction of Ag. Ag will promote the generation and enrichment of ∗CO in the catalyst system, thereby promoting the coupling reaction. A single Ag site is conducive to the enrichment of ∗CO_L_ and further protonation to produce ∗CHO, while the Ag−Ag site is conducive to the enrichment of ∗CO_B_. The combination of the two sites promotes the coupling of ∗CHO and ∗CO_B_ to produce ∗COCHO, which is then converted into ethanol (Figure 9K).

## Summary and outlook

Electrochemical reduction of CO_2_ holds immense promise in addressing energy and environmental concerns by facilitating the synthesis of sustainable fuel ethanol.^117^^,^^118^ However, a significant hurdle lies in the low selectivity for ethanol, mainly due to the favorable formation of competing C_1_ products like CO and methane, as well as C_2+_ products like ethylene.^119^^,^^120^ While minimizing C_1_ products has been successful inhabited through innovative catalyst design and optimized reaction conditions, achieving high ethanol selectivity while effectively suppressing ethylene remains a significant challenge due to the similarity in the reaction pathways leading to the formation of ethylene and ethanol. Researchers have made significant strides in enhancing ethanol efficiency and suppressing ethylene production through innovative catalyst design strategies. One area of exploration has been the manipulation of catalyst surface morphology and reaction environments. By tailoring the morphology and structure of pure metal catalysts, scientists have gained insights into how different surface features can influence the formation of ethanol. Additionally, understanding the Cu valence in catalytic active centers has revealed the potential of oxidation states in promoting ethanol generation. Alloy catalysts have emerged as promising candidates for enhancing ethanol selectivity. Their ability to form multi-metal active centers allows for the generation of asymmetric ethanol products. Furthermore, catalysts based on the concept of tandem catalysis have been designed to promote cooperative reactions and divide labor, effectively boosting ethanol production. Composite catalysts have also been a subject of investigation, as they play a crucial role in improving the interface environment for efficient ethanol synthesis. On the other hand, no-copper catalysts have demonstrated high selectivity for ethanol, presenting exciting opportunities for enol conversion in CO_2_RR. Collectively, these research efforts not only advance our knowledge of the mechanisms behind ethanol formation but also enrich the possibilities for improving the selectivity of CO_2_ electroreduction toward ethanol. While progress has been achieved in the laboratory-scale electrochemical conversion of CO_2_ to ethanol, significant challenges remain for scaling up the process. The first challenge is the need for cost-effective, large-scale preparation of high-selectivity catalysts. Additionally, the currently employed equipment, such as H-type cells and flow cells, faces high voltage requirements, hindering large-scale ethanol production. MEAs are better suited for this purpose, but necessitate the development of catalysts capable of enabling long-term, stable, low-voltage ethanol production in large-area MEA battery stacks. Product separation also presents a key issue. Thus, the development of new catalysts is crucial for realizing industrial-scale electrochemical conversion of CO_2_ to ethanol In Table 1, we comprehensively summarize the key findings from a selection of representative studies. This structured presentation not only enables straightforward horizontal comparison but also facilitates the extraction of pertinent information for further analysis and synthesis. Furthermore, we summarize the mainstream mechanisms of electrocatalytic CO2 reduction to ethanol as follows: (1) concentration of intermediate ∗CO coverage, a high coverage of ∗CO not only decreases the reaction energy for the C−C coupling step, but also steers the selectivity from ethylene to ethanol. The CO spillover strategy in tandem catalysts and alloy catalysts, along with the defect site-enhanced CO adsorption strategy, are both designed based on this theoretical foundation. (2) Asymmetric catalytic center, efficient preparation of ethanol involves constructing asymmetric centers during catalyst design, guided by its structurally asymmetric nature. For example, the copper/gold heterojunction promotes the asymmetrical electrohydrogenation of CO_2_, favoring the production of ethanol over ethylene. By incorporating Ag, the coordinated number and oxide state of surface Cu sites is optimized, leading to ∗CO adsorption in both atop and bridge configurations, facilitating the stabilization of ethanol intermediates through asymmetric C−C coupling. The presence of Cu(I)/Cu(0) in a steady state through a redox shuttle manner enables dual CO molecules to couple asymmetrically, further enhancing the selectivity toward asymmetric C_2+_ products, namely ethanol. (3) Concentration of intermediate H coverage, the elevated ∗H coverage favors the hydrogenation of the ∗HCCOH intermediate, accounting for the increased yield of ethanol.^121^ Recent studies have shown that ∗H, although often associated with the generation of competing product H_2_, plays a crucial role in guiding the efficient conversion of CO_2_ into ethanol. By carefully adjusting the density of Cu surface ∗H, researchers have been able to achieve enhanced selectivity for ethanol production. The mechanism we have summarized represents only a fraction of the numerous insights into converting CO_2_ to ethanol instead of ethylene. Thus, there are still many unique insights that we have not fully summarized. As we continue to enrich our understanding, a more comprehensive and profound comprehension of the entire process will be achieved.

**Table 1:** The list of representative catalysts for ethanol production and their performance are summarized in

Materials	Electrolyte	Cell	Potential (V vs. RHE)	FE_ethanol_ (%)	j_ethanol_ (mA cm^−2^)	Reference
*p*-CuO-(12.5 nm)	3.0 M KOH	Flow cell	−0.87	44.1	501	Wang et al.^61^
dCu_2_O/Ag_2.3%_	1.0 M KOH	MEA Flow cell	−2.11 no iR correction	40.8	326.4	Wang et al.^20^
N-C/Cu	1.0 M KOH	Flow cell	−0.68	52	156	Wang et al.^32^
Ce(OH)x/Cu/PTFE	1.0 M KOH	Flow cell	−0.70	43	128	Liu et al.^98^
Cu/FeTPP [Cl]	1.0 M KHCO_3_	Flow cell	−0.82	41	124	Su et al.^91^
CuS/Cu-V	1.0 M KOH	Flow cell	−0.95	25	100	Liu et al.^41^
Cu_3_Ag_1_	0.5 M KHCO_3_	H-cell	−0.95	44	17	Yang et al.^77^
Ag_0.14_/Cu_0.86_	1.0 M KOH	Flow cell	−0.67	41	250	Wang et al.^78^
F-Cu	1.0 M KOH	Flow cell	−0.89	15	240	Ma et al.^29^
NGQ/Cu-nr	1.0 M KOH	Flow cell	−0.90	45	127	Chen et al.^33^
Cu_5_Zn_8_	0.1 M KHCO_3_	H-cell	−0.80	46	1.84	Peng et al.^84^
Ag/Cu	0.1 M KHCO_3_	H-cell	−1.50	48	3.4	Hoang et al.^28^
Cu_9_Zn_1_/PTFE	1.0 M KOH	MEA	−0.76	25	93	Luo et al.^82^
Cu_500_Ag_1000_	1.0 M KOH	Flow cell	−0.70	30<	120	Ren et al.^95^
MC−CNT/Co	0.5 M KHCO_3_	H-cell	−0.32	60	5.1	Iyengar et al.^101^
Bimetallic AgCu	1.0 M KOH	Flow cell	−0.67	41	102	Wang et al.^78^
Multi-Cu_2_O	2.0 M KOH	Flow cell	−0.61	27	96	Dou et al.^64^
Cu_-DS_	0.1 M KHCO_3_	H-cell	−1.08	53	16	Xiao et al.^42^
	1.0 M KOH	Flow cell	−0.95	52	52	
CuDTA	1.0 M KOH	Flow cell	−0.70	27	75	Ocampo-Restrepo et al.^39^
Cu/Cu_2_O	0.4 M K+	H-cell	−1.1	41.2	36.9	Yang et al.^63^
Cu^+^/hf-Cu	0.1 M KCl	H-cell	−0.8	43	0.23	Lu et al.^62^
Wrinkled Cu	0.1 M KCl	H-cell	−1.1	40	2.2	Santatiwongchai et al.^43^
Cu/Au	1.0 M KOH	Flow cell	−0.75	60	300	Kuang et al.^21^
Ag-Cu_2_O_PB_	0.2 M KCl	H-cell	−1.2	34	1.1	Zeng et al.^80^
CuSn_0.025_	0.1 M KHCO_3_	H-cell	−1.4	25	15	Kim et al.^73^
Cu-Cu_2_S	0.1 M KHCO_3_	H-cell	−1.2	46	20.7	Rahaman et al.^57^
CuO_x_	0.5 M KHCO_3_	H-cell	−0.75	12.9	0.25	Li et al.^68^
CoCu		H-cell		61		Li et al.^85^
Cu nanoparticles	1.0 M KOH	Flow cell	−0.8	17	50	She et al.^107^
200 nm Cu/PTFE	0.1 M KHCO_3_	MEA	−4.7 Tank pressure	20	40	Miao et al.^30^
Au/Cu bimetallic	0.1 M KHCO_3_	H-cell	−0.9	5	0.15	Morales-Guio et al.^31^
Cu_p-ED_(240)/AgIOs	0.2 M KHCO_3_	H-cell	−1.05	33.2	17.5	Liu et al.^93^
Cu Mesh (Cu_2_O)	0.5 M KHCO_3_	H-cell	−1.0	13	1.5	Liu et al.^46^
Cu_x_Zn	0.1 M KHCO_3_	H-cell	−1.05	29.1	8.2	Li et al.^83^
3.6 μm Cu_2_O film/Cu	0.1 M KHCO_3_	H-cell	−0.99	16	5.6	Schouten et al.^47^
c-NC	0.1 M KHCO_3_	H-cell	−0.56	77	0.2	Abeyweera et al.^102^
Cu/N_0.14_C	0.1 M KHCO_3_	H-cell	−1.1	51	14.4	Park et al.^106^
Cu_1.22_V_0.19_Se	1.0 M KOH	Flow cell	−0.80	68.3	207.9	Chen et al.^48^
Cu(Ag-20)_20_	0.1 M KHCO_3_	Flow cell	−1.1	16.5	4.1	Xu et al.^108^
100-cycle Cu	0.25 M KHCO_3_	H-cell	−0.95	13	7.5	Luo et al.^109^
V_Se_-Cu_2-x_Se	0.5 M KHCO_3_	H-cell	−0.80	68.1	7	Hoang et al.^49^
BaO/Cu	1.0 M KOH	Flow cell	−0.75	50	200	Chen et al.^97^
Cu/C-0.4	0.1 M KHCO_3_	Flow cell	−0.70	91	1.23	Xu et al.^25^
Cu/C-0.8	0.1 M KHCO_3_	Flow cell	−0.70	90	1.38	
Hex-2Cu-O	1.0 M KOH	Flow cell	−0.66	25	69	Yang et al.^24^
	0.1 M KHCO_3_	H-cell	−1.20	32.5	3.97	
CuO-FC	1.0 M KOH	Flow cell	−1.0	35.7	127.2	Ren et al.^58^
PGA-2	0.5 M KHCO_3_	H-cell	−0.80	48.7	4.7	Ma et al.^103^
CeO_2_/CuS	1.0 M KOH	Flow cell	−0.80	54	54.2	Gu et al.^52^
Al-Cu/Cu_2_O	1.0 M KOH	Flow cell	−1.0	48.8	271.5	Zhang et al.^67^
CuAl_2_O_4_ /CuO	1.0 M KOH	Flow cell	−1.0	41	82	Cao et al.^99^
B-doped Cu	0.1 M KCl	H-cell	−1.10	27	18.8	Kuo et al.^54^
CuSn_0.025_	0.1 M KHCO_3_	H-cell	−1.40	25.93	15.05	Kim et al.^73^
CuO/SnO_2_	0.5 M KHCO_3_	H-cell	−1.05	39.84	6.79	Reske et al.^50^
3D Cu–CS-GDL	1 M KOH	Flow cell	−0.87	41.4	372.6	Iyengar et al.^100^
SnS_2_/Sn_1_-O_3_G	0.5 M KHCO_3_	H-cell	−0.90	82.5	14.68	Chen et al.^104^
Ag_0.015_In_0.985_ Se_0.734_	0.1 M KHCO_3_	H-cell	−0.60	75.2	13.4	Li eta al.^105^

**Figure 10:**
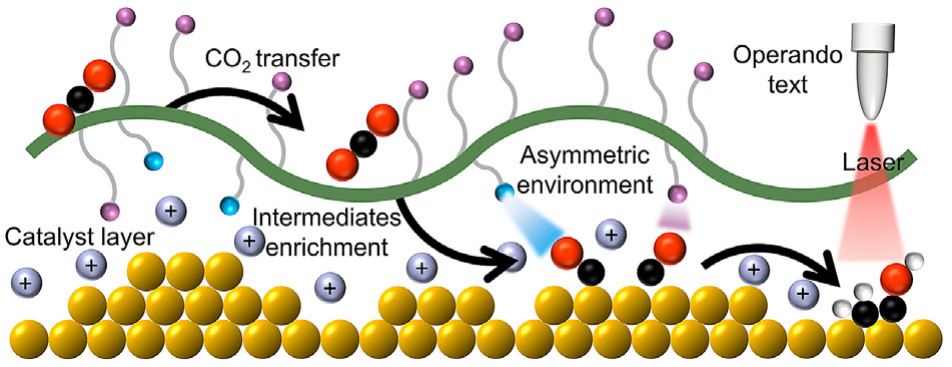
Schematic diagram of the development of an asymmetric environment and the detection of intermediates from CO_2_ reduction to ethanol

To realize a practical CO_2_-to-ethanol electrochemical conversion, it is imperative not only to attain high selectivity but also to fulfill various other crucial performance metrics. These include optimizing the reaction rate, enhancing energy efficiency, ensuring stability, and achieving desired product concentration, as outlined in the provided reference. The theoretical potential for electrochemical CO_2_-to-ethanol conversion is 0.084 V. Assuming perfect efficiency with no performance degradation, the minimum cost of ethanol production via CO_2_ electrolysis is $0.32/L. This is below the 2017 average price on the US Gulf Coast, which was $0.39/L, suggesting that profitable production is feasible. However, these calculations are based on ideal conditions, and actual commercialization faces significant complexities. In catalyst design for ethanol, key considerations include increasing ∗CO coverage and tuning its adsorption strength for C_2_ synthesis. Unlike ethylene, ethanol production relies on stabilizing the C−O bond in oxygen-containing intermediates. Designing catalysts with oxyphilic surfaces aids in preserving these bonds, favoring ethanol over ethylene. Lastly, ∗H coverage on the surface should be optimized to facilitate hydrogenation of intermediates, promoting ethanol generation, while avoiding excessive or insufficient coverage that could favor HER or C_2_H_4_ production. Beyond catalyst design, the CO_2_ to ethanol conversion system also hinges on equipment design. As electrolysis time increases, accumulated ethanol may degrade catalyst performance, necessitating timely product separation. Traditional equipment like flow cells and MEAs struggle with this, prompting the development of solid-state electrolytic cells. While currently used for CO and HCOOH preparation, these could be adapted for ethanol production. Given the presence of other liquid products, achieving high-purity ethanol is crucial, typically involving distillation and molecular sieve filtration. Thus, catalyst design, equipment, and post-treatment are all vital considerations in this. Meeting these ambitious targets demands groundbreaking progress in catalyst design and electrochemical system engineering. The effective enhancement of reactive transfer and increased current density can be achieved through the development and utilization of gas diffusion electrodes (GDEs) and membrane components. However, this progress also brings forth challenges such as product and carbonate/bicarbonate crossover, along with the issue of poor GDE stability. In addition to cell design, exploring the following aspects may help us to create an excellent CO_2_ to ethanol interface: (1) asymmetric reactive microenvironment, In the context of CO_2_RR, the interface microenvironment plays a crucial role in influencing the reaction outcomes. Various factors, such as the local pH value, interface wettability, and cation effects, have been identified as key determinants in significantly improving the selectivity of the products. Taking inspiration from the asymmetric catalytic center in ethanol, innovative approaches could be explored to design electrode materials or catalysts with inherent asymmetry. Such materials may facilitate selective adsorption or catalytic transformations on specific facets or sites, promoting asymmetric product distribution. Combining the inherent asymmetry of ethanol with an asymmetric interfacial microenvironment may hold the key to unlocking new possibilities for achieving higher product selectivity. However, it is essential to carefully optimize and control these asymmetric features to avoid unintended side effects or imbalances that could hinder the overall efficiency of the CO_2_RR process. Moreover, comprehensive understanding and detailed investigations using advanced experimental and computational techniques will be necessary to unravel the underlying mechanisms and ensure successful implementation of this intriguing concept. In conclusion, the potential synergy between the asymmetric characteristics of ethanol and an asymmetric interfacial microenvironment presents an exciting avenue for achieving significant breakthroughs in CO_2_-to-ethanol conversion (Figure 10). 2) Regulation of adsorption intermediates, understanding the role of intermediate species in the CO_2_-to-ethanol conversion is crucial for determining the specific pathway leading to ethanol formation. The current research is limited to a few intermediates like CO and H. To improve the process, further investigation and identification of key intermediates are needed (Figure 10). The use of operando techniques and computational modeling can provide deeper insights into intermediate behavior. Broadening our knowledge of intermediates is essential for advancing CO_2_-to-ethanol conversion.

## References

[1] Fankhauser S., Smith S.M., Allen M., Axelsson K., Hale T., Hepburn C., Kendall J.M., Khosla R., Lezaun J., Mitchell-Larson E. (2022). The meaning of net zero and how to get it right. Nat. Clim. Chang..

[2] Ni Z.Y., Wang P., Quan F., Guo R., Liu C.M., Liu X.W., Mu W.N., Lei X.F., Li Q.J. (2022). Design strategy of a Cu-based catalyst for optimizing the performance in the electrochemical CO_2_ reduction reaction to multicarbon alcohols. Nanoscale.

[3] Li L., Li X.D., Sun Y.F., Xie Y. (2022). Rational design of electrocatalytic carbon dioxide reduction for a zero-carbon network. Chem. Soc. Rev..

[4] Nguyen T.N., Guo J.X., Sachindran A., Li F.W., Seifitokaldani A., Dinh C.T. (2021). Electrochemical CO_2_ reduction to ethanol: from mechanistic understanding to catalyst design. J. Mater. Chem. A.

[5] Gao D.F., Arán-Ais R.M., Jeon H.S., Roldan Cuenya B. (2019). Rational catalyst and electrolyte design for CO_2_ electroreduction towards multicarbon products. Nat. Catal..

[6] Qiao J.L., Liu Y.Y., Hong F., Zhang J.J. (2014). A review of catalysts for the electroreduction of carbon dioxide to produce low-carbon fuels. Chem. Soc. Rev..

[7] Dinh C.T., Burdyny T., Kibria M.G., Seifitokaldani A., Gabardo C.M., de Arquer F.P.G., Kiani A., Edwards J.P., De Luna P., Bushuyev O.S. (2018). CO_2_ electroreduction to ethylene via hydroxide-mediated copper catalysis at an abrupt interface. Science.

[8] Xu D.Z., Li K.K., Jia B.H., Sun W.P., Zhang W., Liu X., Ma T.Y. (2023). Electrocatalytic CO_2_ reduction towards industrial applications. Carbon Energy.

[9] Todorova T.K., Schreiber M.W., Fontecave M. (2020). Mechanistic Understanding of CO_2_ Reduction Reaction (CO_2_RR) Toward Multicarbon Products by Heterogeneous Copper-Based Catalysts. ACS Catal..

[10] Zhang Z.Y., Bian L., Tian H., Liu Y., Bando Y., Yamauchi Y., Wang Z.L. (2022). Tailoring the Surface and Interface Structures of Copper-Based Catalysts for Electrochemical Reduction of CO_2_ to Ethylene and Ethanol. Small.

[11] Kohler M., Basile A., Iulianelli A., Dalena F., Veziroğlu T.N. (2019). Ethanol.

[12] Bushuyev O.S., De Luna P., Dinh C.T., Tao L., Saur G., van de Lagemaat J., Kelley S.O., Sargent E.H. (2018). What Should We Make with CO_2_ and How Can We Make It?. Joule.

[13] Verma S., Kim B., Jhong H., Ma S.C., Kenis P.J.A. (2016). A Gross-Margin Model for Defining Technoeconomic Benchmarks in the Electroreduction of CO_2_. ChemSusChem.

[14] Karapinar D., Creissen C.E., de la Cruz J.G.R., Schreiber M.W., Fontecave M. (2021). Electrochemical CO_2_ Reduction to Ethanol with Copper-Based Catalysts. ACS Energy Lett..

[15] Spurgeon J.M., Kumar B. (2018). A comparative technoeconomic analysis of pathways for commercial electrochemical CO_2_ reduction to liquid products. Energy Environ. Sci..

[16] Lu T.R., Xu T., Zhu S.J., Li J., Wang J.C., Jin H.L., Wang X., Lv J.J., Wang Z.J., Wang S. (2023). Electrocatalytic CO_2_ Reduction to Ethylene: From Advanced Catalyst Design to Industrial Applications. Adv. Mater..

[17] Ouyang Y.X., Shi L., Bai X.W., Ling C.Y., Li Q., Wang J.L. (2023). Selectivity of Electrochemical CO_2_ Reduction toward Ethanol and Ethylene: The Key Role of Surface-Active Hydrogen. ACS Catal..

[18] De Arquer F.P.G., Dinh C.T., Ozden A., Wicks J., McCallum C., Kirmani A.R., Nam D.H., Gabardo C., Seifitokaldani A., Wang X. (2020). CO_2_ electrolysis to multicarbon products at activities greater than 1 A cm^-2^. Science.

[19] Zhong M., Tran K., Min Y.M., Wang C.H., Wang Z.Y., Dinh C.T., De Luna P., Yu Z.Q., Rasouli A.S., Brodersen P. (2020). Accelerated discovery of CO_2_ electrocatalysts using active machine learning. Nature.

[20] Wang P.T., Yang H., Tang C., Wu Y., Zheng Y., Cheng T., Davey K., Huang X.Q., Qiao S.Z. (2022). Boosting electrocatalytic CO_2_-to-ethanol production via asymmetric C−C coupling. Nat. Commun..

[21] Kuang S., Su Y., Li M., Liu H., Chuai H., Chen X., Hensen E.J.M., Meyer T.J., Zhang S., Ma X. (2023). Asymmetrical electrohydrogenation of CO_2_ to ethanol with copper-gold heterojunctions. Proc. Natl. Acad. Sci. USA.

[22] Zheng Y., Vasileff A., Zhou X.L., Jiao Y., Jaroniec M., Qiao S.Z. (2019). Understanding the Roadmap for Electrochemical Reduction of CO_2_ to Multi-Carbon Oxygenates and Hydrocarbons on Copper-Based Catalysts. J. Am. Chem. Soc..

[23] Wang Z., Li Y., Zhao X., Chen S., Nian Q., Luo X., Fan J., Ruan D., Xiong B.Q., Ren X. (2023). Localized Alkaline Environment via In Situ Electrostatic Confinement for Enhanced CO_2_-to-Ethylene Conversion in Neutral Medium. J. Am. Chem. Soc..

[24] Yang B.Y., Chen L., Xue S.L., Sun H., Feng K., Chen Y.F., Zhang X., Xiao L., Qin Y.Z., Zhong J. (2022). Electrocatalytic CO_2_ reduction to alcohols by modulating the molecular geometry and Cu coordination in bicentric copper complexes. Nat. Commun..

[25] Xu H., Rebollar D., He H., Chong L., Liu Y., Liu C., Sun C.-J., Li T., Muntean J.V., Winans R.E. (2020). Highly selective electrocatalytic CO_2_ reduction to ethanol by metallic clusters dynamically formed from atomically dispersed copper. Nat. Energy.

[26] Chen S.H., Ye C.L., Wang Z.W., Li P., Jiang W.J., Zhuang Z.C., Zhu J.X., Zheng X.B., Zaman S., Ou H.H. (2023). Selective CO_2_ Reduction to Ethylene Mediated by Adaptive Small-molecule Engineering of Copper-based Electrocatalysts. Angew. Chem. Int. Ed. Engl..

[27] Liu W., Zhai P.B., Li A.W., Wei B., Si K.P., Wei Y., Wang X.G., Zhu G.D., Chen Q., Gu X.K. (2022). Electrochemical CO_2_ reduction to ethylene by ultrathin CuO nanoplate arrays. Nat. Commun..

[28] Hoang T.T.H., Verma S., Ma S.C., Fister T.T., Timoshenko J., Frenkel A.I., Kenis P.J.A., Gewirth A.A. (2018). Nanoporous Copper Silver Alloys by Additive-Controlled Electrodeposition for the Selective Electroreduction of CO_2_ to Ethylene and Ethanol. J. Am. Chem. Soc..

[29] Ma W.C., Xie S.J., Liu T.T., Fan Q.Y., Ye J.Y., Sun F.F., Jiang Z., Zhang Q.H., Cheng J., Wang Y. (2020). Electrocatalytic reduction of CO_2_ to ethylene and ethanol through hydrogen-assisted C−C coupling over fluorine-modified copper. Nat. Catal..

[30] Miao R.K., Xu Y., Ozden A., Robb A., O'Brien C.P., Gabardo C.M., Lee G., Edwards J.P., Huang J.E., Fan M.Y. (2021). Electroosmotic flow steers neutral products and enables concentrated ethanol electroproduction from CO_2_. Joule.

[31] Morales-Guio C.G., Cave E.R., Nitopi S.A., Feaster J.T., Wang L., Kuhl K.P., Jackson A., Johnson N.C., Abram D.N., Hatsukade T. (2018). Improved CO_2_ reduction activity towards C_2+_ alcohols on a tandem gold on copper electrocatalyst. Nat. Catal..

[32] Wang X., Wang Z.Y., de Arquer F.P.G., Dinh C.T., Ozden A., Li Y.G.C., Nam D.H., Li J., Liu Y.S., Wicks J. (2020). Efficient electrically powered CO_2_-to-ethanol via suppression of deoxygenation. Nat. Energy.

[33] Chen C., Yan X., Liu S., Wu Y., Wan Q., Sun X., Zhu Q., Liu H., Ma J., Zheng L. (2020). Highly Efficient Electroreduction of CO_2_ to C_2+_ Alcohols on Heterogeneous Dual Active Sites. Angew. Chem. Int. Ed. Engl..

[34] Lin Y., Wang T., Zhang L.L., Zhang G., Li L.L., Chang Q.F., Pang Z.F., Gao H., Huang K., Zhang P. (2023). Tunable CO_2_ electroreduction to ethanol and ethylene with controllable interfacial wettability. Nat. Commun..

[35] Zheng Y., Vasileff A., Zhou X., Jiao Y., Jaroniec M., Qiao S.-Z. (2019). Understanding the Roadmap for Electrochemical Reduction of CO_2_ to Multi-Carbon Oxygenates and Hydrocarbons on Copper-Based Catalysts. J. Am. Chem. Soc..

[36] Yan Z., Liu W., Liu X., Shen Z., Li X., Cao D. (2023). Recent Progress in Electrocatalytic Conversion of CO_2_ to Valuable C_2_ Products. Adv. Mater. Interfaces.

[37] Ma W., He X., Wang W., Xie S., Zhang Q., Wang Y. (2021). Electrocatalytic reduction of CO_2_ and CO to multi-carbon compounds over Cu-based catalysts. Chem. Soc. Rev..

[38] Ma Y., Wang J., Yu J., Zhou J., Zhou X., Li H., He Z., Long H., Wang Y., Lu P. (2021). Surface modification of metal materials for high-performance electrocatalytic carbon dioxide reduction. Matter.

[39] Ocampo-Restrepo V.K., Verga L.G., Da Silva J.L.F. (2023). Ab initio study for late steps of CO_2_ and CO electroreduction: from CHCO∗ toward C_2_ products on Cu and CuZn nanoclusters. Phys. Chem. Chem. Phys..

[40] Calle-Vallejo F., Koper M.T.M. (2013). Theoretical Considerations on the Electroreduction of CO to C_2_ Species on Cu(100) Electrodes. Angew. Chem. Int. Ed. Engl..

[41] Liu Z., Song L., Lv X., Liu M., Wen Q., Qian L., Wang H., Wang M., Han Q., Zheng G. (2024). Switching CO_2_ Electroreduction toward Ethanol by Delocalization State-Tuned Bond Cleavage. J. Am. Chem. Soc..

[42] Xiao H., Cheng T., Goddard W.A. (2017). Atomistic Mechanisms Underlying Selectivities in C_1_ and C_2_ Products from Electrochemical Reduction of CO on Cu(111). J. Am. Chem. Soc..

[43] Santatiwongchai J., Faungnawakij K., Hirunsit P. (2021). Comprehensive Mechanism of CO_2_ Electroreduction toward Ethylene and Ethanol: The Solvent Effect from Explicit Water–Cu(100) Interface Models. ACS Catal..

[44] Lum Y., Cheng T., Goddard W.A., Ager J.W. (2018). Electrochemical CO Reduction Builds Solvent Water into Oxygenate Products. J. Am. Chem. Soc..

[45] Bagger A., Ju W., Varela A.S., Strasser P., Rossmeisl J. (2017). Electrochemical CO_2_ Reduction: A Classification Problem. ChemPhysChem.

[46] Liu S., Zhang B.S., Zhang L.H., Sun J. (2022). Rational design strategies of Cu-based electrocatalysts for CO_2_ electroreduction to C_2_ products. J. Energy Chem..

[47] Schouten K.J.P., Calle-Vallejo F., Koper M.T.M. (2014). A Step Closer to the Electrochemical Production of Liquid Fuels. Angew. Chem. Int. Ed. Engl..

[48] Chen C.S., Handoko A.D., Wan J.H., Ma L., Ren D., Yeo B.S. (2015). Stable and selective electrochemical reduction of carbon dioxide to ethylene on copper mesocrystals. Catal. Sci. Technol..

[49] Hoang T.T.H., Ma S.C., Gold J.I., Kenis P.J.A., Gewirth A.A. (2017). Nanoporous Copper Films by Additive-Controlled Electrodeposition: CO_2_ Reduction Catalysis. ACS Catal..

[50] Reske R., Mistry H., Behafarid F., Roldan Cuenya B., Strasser P. (2014). Particle Size Effects in the Catalytic Electroreduction of CO_2_ on Cu Nanoparticles. J. Am. Chem. Soc..

[51] Zhuang T.T., Liang Z.Q., Seifitokaldani A., Li Y., De Luna P., Burdyny T., Che F.L., Meng F., Min Y.M., Quintero-Bermudez R. (2018). Steering post-C−C coupling selectivity enables high efficiency electroreduction of carbon dioxide to multi-carbon alcohols. Nat. Catal..

[52] Gu Z.X., Shen H., Chen Z., Yang Y.Y., Yang C., Ji Y.L., Wang Y.H., Zhu C., Liu J.L., Li J. (2021). Efficient Electrocatalytic CO_2_ Reduction to C_2+_ Alcohols at Defect-Site-Rich Cu Surface. Joule.

[53] Kim J.Y., Park W., Choi C., Kim G., Cho K.M., Lim J., Kim S.J., Al-Saggaf A., Gereige I., Lee H. (2021). High Facets on Nanowrinkled Cu via Chemical Vapor Deposition Graphene Growth for Efficient CO_2_ Reduction into Ethanol. ACS Catal..

[54] Kuo L.K., Dinh C.T. (2021). Toward efficient catalysts for electrochemical CO_2_ conversion to C_2_ products. Curr. Opin. Electrochem..

[55] Duan Y.-X., Meng F.-L., Liu K.-H., Yi S.-S., Li S.-J., Yan J.-M., Jiang Q. (2018). Amorphizing of Cu Nanoparticles toward Highly Efficient and Robust Electrocatalyst for CO_2_ Reduction to Liquid Fuels with High Faradaic Efficiencies. Adv. Mater..

[56] Zhao K., Liu Y.M., Quan X., Chen S., Yu H.T. (2017). CO_2_ Electroreduction at Low Overpotential on Oxide-Derived Cu/Carbons Fabricated from Metal Organic Framework. ACS Appl. Mater. Interfaces.

[57] Rahaman M., Dutta A., Zanetti A., Broekmann P. (2017). Electrochemical Reduction of CO_2_ into Multicarbon Alcohols on Activated Cu Mesh Catalysts: An Identical Location (IL) Study. ACS Catal..

[58] Ren D., Deng Y.L., Handoko A.D., Chen C.S., Malkhandi S., Yeo B.S. (2015). Selective Electrochemical Reduction of Carbon Dioxide to Ethylene and Ethanol on Copper(I) Oxide Catalysts. ACS Catal..

[59] Sun W.P., Wang P., Jiang Y.W., Jiang Z.W., Long R., Chen Z., Song P., Sheng T., Wu Z.C., Xiong Y.J. (2022). V-Doped Cu_2_Se Hierarchical Nanotubes Enabling Flow-Cell CO_2_ Electroreduction to Ethanol with High Efficiency and Selectivity. Adv. Mater..

[60] Wang H.Z., Bi X.Z., Yan Y.F., Zhao Y.Z., Yang Z.X., Ning H., Wu M.B. (2023). Efficient Electrocatalytic Reduction of CO_2_ to Ethanol Enhanced by Spacing Effect of Cu-Cu in Cu_2_Se Nanosheets. Adv. Funct. Mater..

[61] Wang M., Chen H.M., Wang M., Wang J.X., Tuo Y.X., Li W.Z., Zhou S.S., Kong L.H., Liu G.B., Jiang L.H., Wang G.X. (2023). Tuning C_1_/C_2_ Selectivity of CO_2_ Electrochemical Reduction over in-Situ Evolved CuO/SnO_2_ Heterostructure. Angew. Chem. Int. Ed. Engl..

[62] Lu H., Wang G., Zhou Y., Wotango A.S., Wu J.H., Meng Q., Li P. (2022). Concentration Optimization of Localized Cu^0^ and Cu^+^ on Cu-Based Electrodes for Improving Electrochemical Generation of Ethanol from Carbon Dioxide. Int. J. Mol. Sci..

[63] Yang Z., Ji D., Li Z., He Z., Hu Y., Yin J., Hou Y., Xi P., Yan C.-H. (2023). CeO_2_/CuS Nanoplates Electroreduce CO_2_ to Ethanol with Stabilized Cu^+^ Species. Small.

[64] Dou T., Du J.W., He J.Q., Wang Y.P., Zhao X.H., Zhang F.Z., Lei X.D. (2022). Sulfurization-derived Cu^0^-Cu^+^ sites for electrochemical CO_2_ reduction to ethanol. J. Power Sources.

[65] Zhou Y., Che F., Liu M., Zou C., Liang Z., De Luna P., Yuan H., Li J., Wang Z., Xie H. (2018). Dopant-induced electron localization drives CO_2_ reduction to C_2_ hydrocarbons. Nat. Chem..

[66] Lv X.Z., Liu Q., Wang J.H., Wu X.J., Li X.T., Yang Y., Yan J.H., Wu A.J., Wu H.B. (2023). Grain refining enables mixed Cu^+^/Cu^0^ states for CO_2_ electroreduction to C_2+_ products at high current density. Appl. Catal. B Environ..

[67] Zhang R., Chen F.F., Jin H.K., Zhang Y., Hao X.Y., Liu Y.D., Feng T.M., Zhang X.H., Lu Z.M., Wang W.H. (2023). Highly stability Cu plus species in hollow Cu_2_O nanoreactors by modulating cavity size for CO_2_ electroreduction to C_2+_ products. Chem. Eng. J..

[68] Li Y., Chen Y.H., Chen T., Shi G.Q., Zhu L., Sun Y., Yu M. (2023). Insight into the Electrochemical CO_2_-to-Ethanol Conversion Catalyzed by Cu2S Nanocrystal-Decorated Cu Nanosheets. ACS Appl. Mater. Interfaces.

[69] Yang C., Shen H.C., Guan A.X., Liu J.L., Li T.F., Ji Y.L., Al-Enizi A.M., Zhang L.J., Qian L.P., Zheng G.F. (2020). Fast cooling induced grain-boundary-rich copper oxide for electrocatalytic carbon dioxide reduction to ethanol. J. Colloid Interface Sci..

[70] Eilert A., Cavalca F., Roberts F.S., Osterwalder J., Liu C., Favaro M., Crumlin E.J., Ogasawara H., Friebel D., Pettersson L.G.M., Nilsson A. (2017). Subsurface Oxygen in Oxide-Derived Copper Electrocatalysts for Carbon Dioxide Reduction. J. Phys. Chem. Lett..

[71] Zhang J.W., Zeng G.M., Zhu S.Q., Tao H.L., Pan Y., Lai W.C., Bao J., Lian C., Su D., Shao M.H., Huang H.W. (2023). Steering CO_2_ electroreduction pathway toward ethanol via surface-bounded hydroxyl species-induced noncovalent interaction. Proc. Natl. Acad. Sci. USA.

[72] Kim J.Y., Kim G., Won H., Gereige I., Jung W.B., Jung H.T. (2022). Synergistic Effect of Cu_2_O Mesh Pattern on High-Facet Cu Surface for Selective CO_2_ Electroreduction to Ethanol. Adv. Mater..

[73] Kim C., Cho K.M., Park K., Kim J.Y., Yun G.T., Toma F.M., Gereige I., Jung H.T. (2021). Cu/Cu_2_O Interconnected Porous Aerogel Catalyst for Highly Productive Electrosynthesis of Ethanol from CO_2_. Adv. Funct. Mater..

[74] Zhang L., Feng J., Wu L., Ma X., Song X., Jia S., Tan X., Jin X., Zhu Q., Kang X. (2023). Oxophilicity-Controlled CO_2_ Electroreduction to C_2+_ Alcohols over Lewis Acid Metal-Doped Cu^δ+^ Catalysts. J. Am. Chem. Soc..

[75] Lin S.C., Chang C.C., Chiu S.Y., Pai H.T., Liao T.Y., Hsu C.S., Chiang W.H., Tsai M.K., Chen H.M. (2020). Operando time-resolved X-ray absorption spectroscopy reveals the chemical nature enabling highly selective CO_2_ reduction. Nat. Commun..

[76] Cheng T., Xiao H., Goddard W.A. (2017). Full atomistic reaction mechanism with kinetics for CO reduction on Cu(100) from ab initio molecular dynamics free-energy calculations at 298 K. Proc. Natl. Acad. Sci. USA.

[77] Yang P.P., Zhang X.L., Gao F.Y., Zheng Y.R., Niu Z.Z., Yu X.X., Liu R., Wu Z.Z., Qin S., Chi L.P. (2020). Protecting Copper Oxidation State via Intermediate Confinement for Selective CO_2_ Electroreduction to C_2+_ Fuels. J. Am. Chem. Soc..

[78] Wang L.M., Chen W.L., Zhang D.D., Du Y.P., Amal R., Qiao S.Z., Bf J.W., Yin Z.Y. (2019). Surface strategies for catalytic CO_2_ reduction: from two-dimensional materials to nanoclusters to single atoms. Chem. Soc. Rev..

[79] Zhu N., Zhang X., Wang P., Chen N., Zhu J., Zheng X., Chen Z., Sheng T., Wu Z. (2024). Ce^4+^-Doped CuO Mesoporous Nanosheets for CO_2_ Electroreduction to C_2_H_6_ with High Selectivity under a Wide Potential Window in a Flow Cell. ACS Sustainable Chem. Eng..

[80] Zeng S.H., Shan S.Y., Lu A.L., Wang S., Caracciolo D.T., Robinson R.J., Shang G.J., Xue L., Zhao Y.S., Zhang A.A. (2021). Copper-alloy catalysts: structural characterization and catalytic synergies. Catal. Sci. Technol..

[81] Liu L.Z., Akhoundzadeh H., Li M.T., Huang H.W. (2023). Alloy Catalysts for Electrocatalytic CO_2_ Reduction. Small Methods.

[82] Luo M.C., Guo S.J. (2017). Strain-controlled electrocatalysis on multimetallic nanomaterials. Nat. Rev. Mater..

[83] Li P., Liu L., An W., Wang H., Guo H., Liang Y., Cui W. (2020). Ultrathin porous g-C_3_N_4_ nanosheets modified with AuCu alloy nanoparticles and C−C coupling photothermal catalytic reduction of CO_2_ to ethanol. Appl. Catal. B Environ..

[84] Peng Y., Cui M., Zhang Z., Shu S., Shi X., Brosnahan J.T., Liu C., Zhang Y., Godbold P., Zhang X. (2019). Bimetallic composition-promoted electrocatalytic hydrodechlorination reaction on silver–palladium alloy nanoparticles. ACS Catal..

[85] Li M.H., Song N., Luo W., Chen J., Jiang W., Yang J.P. (2023). Engineering Surface Oxophilicity of Copper for Electrochemical CO_2_ Reduction to Ethanol. Adv. Sci..

[86] Lv X., Shang L., Zhou S., Li S., Wang Y., Wang Z., Sham T.-K., Peng C., Zheng G. (2020). Electron-Deficient Cu Sites on Cu_3_Ag_1_ Catalyst Promoting CO_2_ Electroreduction to Alcohols. Adv. Energy Mater..

[87] Li Y.G.C., Wang Z.Y., Yuan T.G., Nam D.H., Luo M.C., Wicks J., Chen B., Li J., Li F.W., de Arguer F.P.G. (2019). Binding Site Diversity Promotes CO_2_ Electroreduction to Ethanol. J. Am. Chem. Soc..

[88] Clark E.L., Hahn C., Jaramillo T.F., Bell A.T. (2017). Electrochemical CO_2_ Reduction over Compressively Strained CuAg Surface Alloys with Enhanced Multi-Carbon Oxygenate Selectivity. J. Am. Chem. Soc..

[89] Ye Y.F., Qian J., Yang H., Su H.Y., Lee K.J., Etxebarria A., Cheng T., Xiao H., Yano J.K., Goddard W.A., Crumlin E.J. (2020). Synergy between a Silver-Copper Surface Alloy Composition and Carbon Dioxide Adsorption and Activation. ACS Appl. Mater. Interfaces.

[90] Kim C., Dionigi F., Beermann V., Wang X.L., Möller T., Strasser P. (2019). Alloy Nanocatalysts for the Electrochemical Oxygen Reduction (ORR) and the Direct Electrochemical Carbon Dioxide Reduction Reaction (CO_2_RR). Adv. Mater..

[91] Su X.S., Sun Y.M., Jin L., Zhang L., Yang Y., Kerns P., Liu B., Li S.Z., He J. (2020). Hierarchically porous Cu/Zn bimetallic catalysts for highly selective CO_2_ electroreduction to liquid C_2_ products. Appl. Catal. B Environ..

[92] Lee S., Park G., Lee J. (2017). Importance of Ag-Cu Biphasic Boundaries for Selective Electrochemical Reduction of CO_2_ to Ethanol. ACS Catal..

[93] Liu S.H., Yang C.S., Zha S.J., Sharapa D., Studt F., Zhao Z.J., Gong J.L. (2022). Moderate Surface Segregation Promotes Selective Ethanol Production in CO_2_ Hydrogenation Reaction over CoCu Catalysts. Angew. Chem. Int. Ed. Engl..

[94] Baek Y., Song H., Hong D., Wang S., Lee S., Joo Y.C., Lee G.D., Oh J. (2022). Electrochemical carbon dioxide reduction on copper-zinc alloys: ethanol and ethylene selectivity analysis. J. Mater. Chem. A.

[95] Ren D., Ang B.S.H., Yeo B.S. (2016). Tuning the Selectivity of Carbon Dioxide Electroreduction toward Ethanol on Oxide-Derived Cu_x_Zn Catalysts. ACS Catal..

[96] Wei C., Yang Y., Ma H., Sun G., Wang X., Cheng Y., Zhang C., Yeo B.S., He C., Wong A.B. (2023). Nanoscale Management of CO Transport in CO_2_ Electroreduction: Boosting Faradaic Efficiency to Multicarbon Products via Nanostructured Tandem Electrocatalysts. Adv. Funct. Mater..

[97] Chen Y.J., Li X.Y., Chen Z., Ozden A., Huang J.E., Ou P.F., Dong J.C., Zhang J.Q., Tian C., Lee B.H. (2024). Efficient multicarbon formation in acidic CO_2_ reduction via tandem electrocatalysis. Nat. Nanotechnol..

[98] Liu M., Wang Q.Y., Luo T., Herran M., Cao X.Y., Liao W.R., Zhu L., Li H.M., Stefancu A., Lu Y.R. (2024). Potential Alignment in Tandem Catalysts Enhances CO_2_-to-C_2_H_4_ Conversion Efficiencies. J. Am. Chem. Soc..

[99] Cao B., Li F.Z., Gu J. (2022). Designing Cu-Based Tandem Catalysts for CO_2_ Electroreduction Based on Mass Transport of CO Intermediate. ACS Catal..

[100] Iyengar P., Kolb M.J., Pankhurst J.R., Calle-Vallejo F., Buonsanti R. (2021). Elucidating the Facet-Dependent Selectivity for CO_2_ Electroreduction to Ethanol of Cu-Ag Tandem Catalysts. ACS Catal..

[101] Iyengar P., Kolb M.J., Pankhurst J., Calle-Vallejo F., Buonsanti R. (2021). Theory-Guided Enhancement of CO_2_ Reduction to Ethanol on Ag-Cu Tandem Catalysts via Particle-Size Effects. ACS Catal..

[102] Abeyweera S.C., Simukaitis M., Wei Q.L., Sun Y.G. (2022). Interfaced Ag/Cu nanostructures derived from metal thiolate nanoplates: A highly selective catalyst for electrochemical reduction of CO_2_ to ethanol. SmartMat.

[103] Ma J.M., Liu C.M., Bai M., Fu Z.M., Zhao P.P., Gao Y., Zhao M., He Y.L., Xiao H., Jia J.F. (2023). Recent advances in application of tandem catalyst for electrocatalytic CO_2_ reduction. Mol. Catal..

[104] Chen C.B., Li Y.F., Yu S., Louisia S., Jin J.B., Li M.F., Ross M.B., Yang P.D. (2020). Cu-Ag Tandem Catalysts for High-Rate CO_2_ Electrolysis toward Multicarbons. Joule.

[105] Li F.W., Li Y.G.C., Wang Z.Y., Li J., Nam D.H., Lum Y., Luo M.C., Wang X., Ozden A., Hung S.F. (2019). Cooperative CO_2_-to-ethanol conversion via enriched intermediates at molecule-metal catalyst interfaces. Nat. Catal..

[106] Park J., Jeong C., Na M., Oh Y., Lee K.-S., Yang Y., Byon H.R. (2024). Subnanometer Cu Clusters on Porous Ag Enhancing Ethanol Production in Electrochemical CO_2_ Reduction. ACS Catal..

[107] She X.J., Zhang T.Y., Li Z.Y., Li H.M., Xu H., Wu J.J. (2020). Tandem Electrodes for Carbon Dioxide Reduction into C_2_ Products at Simultaneously High Production Efficiency and Rate. Cell Rep. Phys. Sci..

[108] Xu A.N., Hung S.F., Cao A., Wang Z.B., Karmodak N., Huang J.E., Yan Y., Rasouli A.S., Ozden A., Wu F.Y. (2022). Copper/alkaline earth metal oxide interfaces for electrochemical CO_2_-to-alcohol conversion by selective hydrogenation. Nat. Catal..

[109] Luo M.C., Wang Z.Y., Li Y.G.C., Li J., Li F.W., Lum Y.W., Nam D.H., Chen B., Wicks J., Xu A.N. (2019). Hydroxide promotes carbon dioxide electroreduction to ethanol on copper via tuning of adsorbed hydrogen. Nat. Commun..

[110] Zhang T.T., Yuan B.W., Wang W.L., He J., Xiang X. (2023). Tailoring H Intermediate Coverage on the CuAl_2_O_4_/CuO Catalyst for Enhanced Electrocatalytic CO_2_ Reduction to Ethanol. Angew. Chem. Int. Ed. Engl..

[111] Bi J.H., Li P.S., Liu J.Y., Jia S.Q., Wang Y., Zhu Q.G., Liu Z.M., Han B.X. (2023). Construction of 3D copper-chitosan-gas diffusion layer electrode for highly efficient CO_2_ electrolysis to C_2+_ alcohols. Nat. Commun..

[112] Du J., Li S.P., Liu S.L., Xin Y., Chen B.F., Liu H.Z., Han B.X. (2020). Selective electrochemical reduction of carbon dioxide to ethanol via a relay catalytic platform. Chem. Sci..

[113] Song Y.F., Chen W., Zhao C.C., Li S.G., Wei W., Sun Y.H. (2017). Metal-Free Nitrogen-Doped Mesoporous Carbon for Electroreduction of CO_2_ to Ethanol. Angew. Chem. Int. Ed. Engl..

[114] Yang F.Q., Liang C.H., Yu H.M., Zeng Z.L., Lam Y.M., Deng S.G., Wang J. (2022). Phosphorus-Doped Graphene Aerogel as Self-Supported Electrocatalyst for CO_2_-to-Ethanol Conversion. Adv. Sci..

[115] Ding J., Bin Yang H., Ma X.L., Liu S., Liu W., Mao Q., Huang Y.Q., Li J., Zhang T., Liu B. (2023). A tin-based tandem electrocatalyst for CO_2_ reduction to ethanol with 80% selectivity. Nat. Energy.

[116] Wang X.Y., Jiang Z.W., Wang P., Chen Z., Sheng T., Wu Z.C., Xiong Y.J. (2023). Ag^+^-Doped InSe Nanosheets for Membrane Electrode Assembly Electrolyzer toward Large-Current Electroreduction of CO_2_ to Ethanol. Angew. Chem. Int. Ed. Engl..

[117] Su X.Z., Jiang Z.L., Zhou J., Liu H.J., Zhou D.N., Shang H.S., Ni X.M., Peng Z., Yang F., Chen W.X. (2022). Complementary Operando Spectroscopy identification of in-situ generated metastable charge-asymmetry Cu_2_-CuN_3_ clusters for CO_2_ reduction to ethanol. Nat. Commun..

[118] Ma S.C., Sadakiyo M., Luo R., Heima M., Yamauchi M., Kenis P.J.A. (2016). One-step electrosynthesis of ethylene and ethanol from CO_2_ in an alkaline electrolyzer. J. Power Sources.

[119] Ting L.R.L., Piqué O., Lim S.Y., Tanhaei M., Calle-Vallejo F., Yeo B.S. (2020). Enhancing CO_2_ Electroreduction to Ethanol on Copper-Silver Composites by Opening an Alternative Catalytic Pathway. ACS Catal..

[120] Jiang K., Sandberg R.B., Akey A.J., Liu X., Bell D.C., Nørskov J.K., Chan K., Wang H. (2018). Metal ion cycling of Cu foil for selective C−C coupling in electrochemical CO_2_ reduction. Nat. Catal..

[121] Li J., Xu A.N., Li F.W., Wang Z.Y., Zou C.Q., Gabardo C.M., Wang Y.H., Ozden A.N., Xu Y., Nam D.E.Y. (2020). Enhanced multi-carbon alcohol electroproduction from CO via modulated hydrogen adsorption. Nat. Commun..

